# Ablation of neuropsin–neuregulin 1 signaling imbalances ErbB4 inhibitory networks and disrupts hippocampal gamma oscillation

**DOI:** 10.1038/tp.2017.20

**Published:** 2017-03-07

**Authors:** M Kawata, S Morikawa, S Shiosaka, H Tamura

**Affiliations:** 1 Laboratory of Functional Neuroscience, Graduate School of Biological Sciences, Nara Institute of Science and Technology (NAIST), Nara, Japan; 2 Department of Pharmacology, Hoshi University School of Pharmacy and Pharmaceutical Sciences, Tokyo, Japan; 3 Life Science Tokyo Advanced Research Center (L-StaR), Hoshi University School of Pharmacy and Pharmaceutical Sciences, Tokyo, Japan

## Abstract

Parvalbumin-expressing interneurons are pivotal for the processing of information in healthy brain, whereas the coordination of these functions is seriously disrupted in diseased brain. How these interneurons in the hippocampus participate in pathological functions remains unclear. We previously reported that neuregulin 1 (NRG1)–ErbB4 signaling, which is actuated by neuropsin, is important for coordinating brain plasticity. Neuropsin cleaves mature NRG1 (bound to extracellular glycosaminoglycans) in response to long-term potentiation or depression, liberating a soluble ligand that activates its receptor, ErbB4. Here, we show in mice that kainate-induced status epilepticus transiently elevates the proteolytic activity of neuropsin and stimulates cFos expression with a time course suggesting that activation of ErbB4- and parvalbumin-expressing interneurons follows the excitation and subsequent silencing of pyramidal neurons. In neuropsin-deficient mice, kainate administration impaired signaling and disrupted the neuronal excitation–inhibition balance (E/I balance) in hippocampal networks, by decreasing the activity of parvalbumin-positive interneurons while increasing that of pyramidal neurons, resulting in the progression of status epilepticus. Slow, but not fast, gamma oscillations in neuropsin-deficient mice showed reduced power. Intracerebroventricular infusion of the soluble NRG1 ligand moiety restored the E/I balance, status epilepticus and gamma oscillations to normal levels. These results suggest that the neuropsin–NRG1 signaling system has a role in pathological processes underlying temporal lobe epilepsy by regulating the activity of parvalbumin-expressing interneurons, and that neuropsin regulates E/I balance and gamma oscillations through NRG1–ErbB4 signaling toward parvalbumin-expressing interneurons. This neuronal system may be a useful target of pharmacological therapies against cognitive disorders.

## Introduction

Cognition and its dysfunction are thought to depend on the coordinated excitation–inhibition (E/I) balance controlled by inhibitory inputs on the principal neurons in neural networks.^1^ Evidence suggests that parvalbumin-containing GABAergic inhibitory neurons are required for the synchronization of neural activities by oscillations in the hippocampus and cerebral cortex.^2^ In particular, parvalbumin-positive fast-spiking neurons, the major population of basket interneurons, coordinate the E/I balance and generate theta and gamma oscillations;^3, 4, 5, 6^ both of these actions are involved in attention, memory and executive functions.^7^ Impairment of parvalbumin-expressing interneuron function leads to seizures^8^ and asynchronous network activity,^3^ thereby contributing to cognitive disturbance, which is the core feature of schizophrenia.^9^ As such, parvalbumin-expressing interneuron signaling is of utmost importance in cognitive brain functions based on the precise synchronization of neuronal ensembles. However, the mechanisms by which parvalbumin-expressing interneurons participate in active network communications are not well understood.

Limited proteolysis of trophic or signaling proteins at synapses induces plastic changes in neural function by irreversibly converting a precursor protein into a biologically active form.^10^ Because the activation of synaptic proteolysis largely depends on neural activity, extracellular proteases have distinct effects on activity-dependent neural events that underlie cognitive function.^11, 12, 13^ Indeed, recent studies showed that extracellular proteolysis has a critical role in dynamic synaptic remodeling, long-term potentiation, synaptic plasticity, memory formation and epileptogenesis.^14, 15, 16, 17, 18, 19^

The hippocampus and amygdala, two brain regions that show a considerable degree of neural plasticity in the adult brain, express the extracellular serine protease neuropsin.^20^ Neuropsin is synthesized as a zymogen, which is then secreted and stored in the extracellular space in an inactive precursor form^21^ that is transiently converted to an active form during neural activity;^22^ this then has a role in long-term potentiation, working memory, epileptogenic insult and anxiety.^15, 23, 24^ Because single nucleotide polymorphisms in the human neuropsin gene are associated with attention and concentration, verbal IQ disorders and bipolar disorder,^25^ neuropsin has recently garnered attention as an attractive target for therapeutic intervention.

We recently reported that, in the mouse hippocampus, neuropsin cleaves neuregulin 1 (NRG1) within a synaptic signaling system.^26^ NGR1 is a neurotrophic factor belonging to the epidermal growth factor family and exists in six isoforms.^27^ Among these, type I, II and II are mainly expressed in the hippocampus of adult mice.^28^ They are transmembrane proteins and are cleaved by various proteases to release (except in the case of NRG1 type III) a soluble extracellular domain called mature NRG1 or mNRG1.^27^ In particular, owing to its heparin-binding domain, mNRG1 type I is confined to the glycosaminoglycan component of the extracellular matrix; however, upon processing by neuropsin it diffuses across the synapse and binds to its receptor, ErbB4.^26^ ErbB4 is expressed specifically by GABAergic neurons, particularly parvalbumin-expressing interneurons;^29^ thus proteolytic function of neuropsin is to specifically activate GABAergic neurons. Indeed, neuropsin-knockout (KO) mice show specific impairment of GABA_A_ receptor-mediated transmission, which can be recovered by hippocampal application of a soluble form of NRG1 comprising its ligand domain.^26^ These findings strongly suggest that neuropsin regulates the activity of parvalbumin-positive GABAergic neurons, particularly ErbB4-containing basket interneurons, via processing of mNRG1. Here, we examined the contribution of neuropsin–NRG1 signaling to the pathological functions of ErbB4-expressing, parvalbumin-positive GABAergic interneurons in the kainate (KA)-induced status epilepticus mouse model. We also examined whether neuropsin contributes to the E/I balance of local networks by counting the number of active pyramidal neurons and ErbB4-expressing parvalbumin-positive interneurons that were immunoreactive for c-Fos.

## Materials and methods

### Animals

A total of 261 adult male C57BL/6 J mice (aged 6–10 weeks; SLC, Hamamatsu, Japan) and 82 neuropsin-KO mice and corresponding 70 wild-type mice (aged 6–10 weeks) were used in this study. Neuropsin-KO mice were generated as previously described^30^ and backcrossed into the C57BL/6 J background for at least 20 generations. Animals were housed in cages with a 12 h light/dark cycle (lights on at 0800 hours) and given food and water ad libitum. All procedures conformed to the US National Research Council's Guide for the Care and Use of Laboratory Animals and were approved by the Nara Institute of Science and Technology's Animal Care and Use Committee. The procedures employed minimized animal suffering, and the lowest number of animals needed to produce the required results was used.

### Electrophysiology

Mice were anesthetized with urethane (1.25 g kg^−1^; intraperitoneal (i.p.)) and placed in a stereotaxic frame (Narishige, Tokyo, Japan). A tungsten recording microelectrode (MicroProbes, Gaithersburg, MD, USA) was implanted into the right hippocampal CA1 pyramidal cell layer (2.46 mm posterior to the bregma, 2 mm lateral to the midline and 1.20 mm ventral to the dural surface). To generate gamma oscillations, KA (25 mg kg^−1^; i.p.) was injected only once. The local field potential was recorded for at least 30 min before and 4 h after KA administration. The power spectra and integral power for the slow gamma (30–50 Hz) and the fast gamma (65–120 Hz) range of the local field potential were calculated in 5 min segments across the recording using the fast Fourier transform algorithm in LabChart software (ADInstruments, Dunedin, New Zealand). The position of the recording site was verified histologically.

### KA administration and behavioral seizure scoring

Status epilepticus was induced according to the method of Sperk *et al.*^31^ Briefly, mice received an i.p. injection of KA (25 mg kg^−1^; Enzo Life Sciences, Farmingdale, NY, USA) dissolved in phosphate-buffered saline, pH 7.4 (PBS; Nacalai Tesque, Kyoto, Japan), and were killed 1, 2, 3, 4, 6, 8 and 24 h later. Control animals received an equivalent volume of PBS. Seizure severity was scored on Racine's scale^32^ modified for mice, as follows: stage 0, normal activity; stage 1, immobility and staring; stage 2, forelimb or tail extension, rigid posture; stage 3, repetitive movements, head bobbing; stage 4, rearing and falling, hindlimb tonic-clonic movements; stage 5, continuous tonic-clonic seizures with running or jumping; and stage 6, death. Only mice that displayed stage 4 or 5 seizure activity were used in this study unless otherwise specified. Additional low doses of KA (10 mg kg^−1^; i.p.) were administered to mice that did not exhibit a continuous motor seizure (stage 4 or 5) within 1 h of the first KA injection.

### Sample preparation

Mice were killed by decapitation at various times after the administration of KA. Whole hippocampi were dissected on an ice-cold surface, frozen on dry ice and then stored at −80 °C until use. The hippocampal tissue was homogenized using 10 strokes of a glass-Teflon homogenizer (Taiyo, Osaka, Japan) with a motor-driven pestle.

### Measurement of neuropsin protein and its proteolytic activity

Measurement of the total amount and proteolytic activity of neuropsin was conducted as described previously.^33, 34^ Briefly, hemi-hippocampal tissue from each mouse was homogenized in 0.75 ml of lysis buffer (50 mm HEPES, pH 7.5, 150 mm NaCl, 5 mm EDTA, 1% Triton X-100 and 5 μg ml^−1^ leupeptin) and then centrifuged at 14 000 × *g* for 30 min at 4 °C to remove debris. The supernatant was incubated with the rat monoclonal anti-mouse neuropsin antibody mAbF12 (0.1 μg; Molecular and Biological Laboratories, Nagoya, Japan) overnight at 4 °C. The immunoprecipitated neuropsin was incubated with the synthetic fluorescent substrate Pro-Phe-Arg-4-methyl-coumaryl-7-amide (Peptide Institute, Osaka, Japan) at 37 °C for 18 h. The product (7-amino-4-methylcoumarin) was quantified using a fluorescence spectrophotometer (F-4500; Hitachi, Tokyo, Japan). Neuropsin protease activity was calculated from a standard curve that was generated by serially diluting aliquots of recombinant neuropsin.^21^ Data were normalized to PBS-injected controls.

### Production of mNRG1-Fc protein

A fragment of mouse *Nrg1 type I* encoding mNRG1 (ref. 26) was cloned into pFUSE-rIgG-Fc2 (IL2ss) (Nacalai Tesque) in frame with the Fc portion of the rabbit Ig. The fused mouse *mNrg1-Fc* gene was then inserted into pGEX-6P-1 (GE Healthcare Life Sciences, Tokyo, Japan). Recombinant glutathione-*S*-transferase*-*mouse-mNRG1*-*Fc was expressed in *Escherichia coli* and captured with glutathione Sepharose (GE Healthcare). Mouse mNRG1*-*Fc was eluted by cleavage of the glutathione-*S*-transferase tag with PreScission Protease (GE Healthcare). The correct sizes of mouse mNRG1*-*Fc and control Fc protein were verified by western blotting with a horseradish peroxidase (HRP)-conjugated donkey anti-Fc antibody (1:10 000, Jackson ImmunoResearch Laboratories, West Grove, PA, USA).

### Immunocytochemistry in MCF-7 cells

Human breast cancer MCF-7 cells (5 × 10^4^ cells per well; JCRB0134, JCRB Cell Bank, National Institutes of Biomedical Innovation, Health and Nutrition, Osaka, Japan) were plated in 24-well plates in high-glucose DMEM with l-glutamine (Sigma-Aldrich, St. Louis, MO, USA) supplemented with 10% fetal bovine serum, 100 U ml^−1^ penicillin and 100 μg ml^−1^ streptomycin at 37 °C in 5% CO_2_. The culture medium was changed to Opti-MEM (Life Technologies, Carlsbad, CA, USA) 24 h after seeding. An additional 24 h later, cells were treated with Fc protein or mouse mNRG1*-*Fc with or without 10 mg ml^−1^ heparin (Sigma-Aldrich) or neuropsin (10 mU ml^−1^) in the culture medium for 60 min, washed and then incubated with an Alexa 488-conjugated donkey anti-rabbit IgG in fresh Opti-MEM (A21206; 1:1000, Molecular Probes, Eugene, OR, USA) for 1.5 h at room temperature. The cells were mounted in ProLong Diamond with DAPI (Life Technologies). Images were captured using a Zeiss LSM710 confocal laser-scanning microscope (Carl Zeiss MicroImaging, Jena, Germany). Fluorescence intensity was measured with ImageJ software (version 1.48; National Institutes of Health, Bethesda, MD, USA).

### Cleavage of mNRG1 by neuropsin

Recombinant human mNRG1 type I (amino acids 2–246; R&D Systems, Minneapolis, MN, USA) was incubated with neuropsin (10 mU ml^−1^) at 37 °C for 60 min in reaction buffer (50 mm Tris–HCl, pH 8 and 0.5 mm CaCl_2_). Cleaved human mNRG1 was separated on 15% SDS-PAGE gels and immunoblotted with a rabbit polyclonal anti-C-terminal mNRG1 antibody (H-210; 1:400, Santa Cruz Biotechnology, Dallas, TX, USA). The blots were developed with an HRP-conjugated donkey anti-rabbit IgG antibody (1:10,000, Jackson ImmunoResearch Laboratories) and chemiluminescent (Immobilon Western; Millipore, Billerica, MA, USA).

### mNRG1-induced receptor phosphorylation assay

The receptor phosphorylation assay was performed as previously described.^26^ Neuropsin (10 mU ml^−1^) or vehicle was incubated with 0–16 nm human mNRG1 at 37 °C for 60 min and then the mixture applied to MCF-7 cells. After 20 min, cells were lysed with lysis buffer (50 mm HEPES, pH 7.5, 150 mm NaCl, 1% Triton X-100, 1% CHAPS, 5 mm EDTA, 50 mm sodium fluoride, 1 mm PMSF, 10 mm sodium pyrophosphate, 1 mm sodium orthovanadate and 1% protease inhibitor cocktail (P8340; Sigma-Aldrich)). The lysates were centrifuged at 14 000 × *g* for 10 min. Protein concentrations in the supernatants were determined with a BCA protein assay kit (Thermo Fisher Scientific, Waltham, MA, USA), and equal amounts of total protein were subjected to 7.5% SDS-PAGE and transferred to nitrocellulose membranes (Bio-Rad Laboratories, Hercules, CA, USA). Membranes were blocked with 5% skimmed milk prepared in Tris-buffered saline containing 0.1% Tween-20 for 30 min. Next, the membranes were probed with a mouse monoclonal anti-phosphotyrosine antibody (4G10; 1:1,000, Millipore) and an HRP-conjugated mouse monoclonal anti-β-actin antibody (ab20272; 1:40 000, Abcam, Cambridge, UK) overnight at 4 °C, followed by a second incubation with an HRP-conjugated donkey anti-rabbit IgG antibody (1:10 000, Jackson ImmunoResearch Laboratories) for 1 h at room temperature. After incubation with a chemiluminescent reagent (Immobilon Western; Millipore), the membranes were exposed to X-ray films (Fujifilm, Tokyo, Japan).

### Quantitative real-time PCR

Total RNA was extracted from hippocampi using TRIzol reagent (Life Technologies) according to the manufacturer's instructions. For cDNA synthesis, total RNA (0.5 μg) was reverse transcribed with TaqMan reverse transcription reagents using random hexamers (Life Technologies). Quantitative real-time PCR was conducted using a LightCycler Instrument (Roche Diagnostics, Basel, Switzerland) with SYBR Green PCR Master Mix (Roche Applied Science, Upper Bavaria, Germany). Following denaturation for 10 min at 95 °C, the reactions were cycled 40 times with denaturation at 95 °C for 10 s, annealing at 60 °C for 10 s and elongation at 72 °C for 14 s. The following primers were used to amplify specific cDNA regions of interest: *Nrg1 type I* (sense, 5′-ATGTCTGAGCGCAAAGAAGG-3′ antisense, 5′-CTCCTGGCTTTTCATCTCTTTCA-3′), neuropsin gene *Klk8* (sense, 5′-CCCACTGCAAAAAACAGAAG-3′ antisense, 5′-TGTCAGCTCCATTGCTGCT-3′) and *Gapdh* (sense, 5′-CGGGAAGCCCATCACCATC-3′ antisense, 5′-GAGGGGCCATCCACAGTCTT-3′).

### NRG1 ELISA

NRG1 protein levels were measured using a commercial sandwich ELISA kit according to the manufacturer's instructions (Uscn Life Science, Hubei, People's Republic of China). Briefly, the hippocampal tissues were homogenized in a motor-driven glass-Teflon homogenizer with PBS containing 1% Triton X-100 and 1% protease inhibitor cocktail, followed by centrifugation at 14 000 × *g* for 10 min at 4 °C. The supernatants were collected and aliquoted into 96-well plates pre-coated with an antibody specific to NRG1, and the plate was incubated for 2 h at 37 °C. A biotin-conjugated antibody specific to NRG1 was added and incubated, followed by the addition of avidin-conjugated HRP. Tetramethylbenzidine was used as the chromogenic substrate. The optical density was read with a microplate reader at 450 nm (Bio-Rad Laboratories), and the concentrations of NRG1 were calculated from a standard curve of purified NRG1 provided with the kit.

### Western blot analysis

Mice were killed by decapitation, and the hippocampal region was rapidly dissected. The tissue was suspended in ice-cold isotonic sucrose buffer (50 mm HEPES, pH 7.5, 0.32 m sucrose, 5 mm EDTA, 1 mm sodium fluoride, 1 mm sodium orthovanadate, and 1% protease inhibitor cocktail) and homogenized on ice in a glass-Teflon homogenizer. Subsequent procedures were performed at 4 °C. Homogenates were centrifuged at 1000 × *g* for 5 min to remove nuclei. The supernatants were centrifuged again at 10 000 × *g* for 20 min to pellet the crude membrane fraction. The obtained membrane preparations were lysed in lysis buffer (5 mm HEPES, pH 7.5, 150 mm NaCl, 5 mm EDTA, 1% SDS, 1 mm sodium fluoride, 1 mm sodium orthovanadate, and 1% protease inhibitor cocktail), and the lysates were centrifuged at 14 000 × *g* to remove debris. The protein concentrations of the supernatants were determined with a BCA protein assay kit (Thermo Fisher Scientific), and equal amounts of total protein were separated on 7.5% SDS-PAGE gels. Following transfer to nitrocellulose membranes (Bio-Rad Laboratories), free protein-binding sites were blocked with 5% skimmed milk prepared in Tris-buffered saline containing 0.1% Tween-20 for 30 min. The membranes were incubated with rabbit monoclonal anti-phospho-ErbB4 (Y1284) (21A9; 1:1,000, Cell Signaling Technology, Danvers, MA, USA) and HRP-conjugated mouse monoclonal anti-β-actin (ab20272; 1:40 000, Abcam) antibodies overnight at 4 °C. Subsequently, the blots were developed using an HRP-conjugated donkey anti-rabbit IgG antibody (1:10 000, Jackson ImmunoResearch Laboratories) and the chemiluminescent reagent (Immobilon Western; Millipore), and exposed to X-ray films (Fujifilm). The blots were stripped and hybridized with a rabbit polyclonal anti-C-terminal ErbB4 antibody (C-18) (sc-283; 1:600, Santa Cruz Biotechnology) to determine the total amount of ErbB4. Band densities were quantified with ImageJ software. The staining of β-actin was used as a standard for protein quantification.

### Immunohistochemical analysis of hippocampal tissue

Mice were anesthetized with urethane (1.25 g kg^−1^; i.p.) 1 or 4 h after KA administration. They were transcardially perfused with 0.85% sterile saline and then with 4% paraformaldehyde prepared in PBS, followed by post-fixation overnight at 4 °C. The brains were cryoprotected in 30% sucrose solution at 4 °C for 48 h and then sectioned coronally at a thickness of 30 μm using a sliding microtome. After blocking with 5% BSA prepared in PBS for 1 h, the sections were incubated overnight at 4 °C with the following antibodies: rabbit polyclonal anti-cFos (AB-5; 1:5000, Millipore), goat polyclonal anti-cFos (sc-52-G; 1:1000, Santa Cruz Biotechnology), a rabbit polyclonal anti-C-terminal ErbB4 antibody (C-18) (sc-283; 1:100, Santa Cruz Biotechnology), mouse monoclonal anti-ErbB4 (Ab77) (H4.77.16; 1:100, Novus Biologicals, Littleton, CO, USA), mouse monoclonal anti-CAMKII (Clone 6G9; 1:100, Millipore), rabbit polyclonal anti-parvalbumin (ab11427; 1:3000, Abcam) and mouse monoclonal anti-parvalbumin (P3088; 1:3000, Sigma-Aldrich). The following secondary antibodies used in the study were all purchased from Thermo Fisher Scientific: Alexa 488-conjugated donkey anti-mouse IgG (1:1000; A21203), Alexa 488-conjugated donkey anti-rabbit IgG (1:1000; A21206), Alexa 594-conjugated donkey anti-mouse IgG (1:1000; A21203), Alexa 594-conjugated donkey anti-rabbit IgG (1:1000; A21207), Alexa 594-conjugated donkey anti-goat IgG (1:1000; A11058), Alexa 647-conjugated donkey anti-mouse IgG (1:1000; A31571), Alexa 647-conjugated donkey anti-rabbit IgG (1:1000; A31573), and Alexa 647-conjugated donkey anti-goat IgG (1:1000; A21447). Nuclei were labeled with Hoechst 33342 dye (Dojindo Laboratories, Kumamoto, Japan). Stained sections were mounted using Prolong Gold antifade reagent (Life Technologies).

### Cell counting analysis

Six sequential sections were imaged at 2 μm intervals along the *z*-axis using a Zeiss confocal microscope fitted with a × 20 objective lens. The cFos-, ErbB4- and parvalbumin-positive cells (that is, cells showing fluorescence intensity above background) in the hippocampal CA1 region were estimated by disector-based cell counting method using ImageJ software as described earlier.^35^ The pairs of parallel sections were set and only neurons that appear in one of the two sections (reference section) and not in the other (look-up section) were counted (Supplementary Figure 1). The average value per unit area (500 000 μm^3^) in five slices from each animal was calculated. To avoid any bias, stereological examination was performed by an investigator blinded to the genotype. The fraction of pyramidal neurons, ErbB4-positive neurons, and parvalbumin-positive interneurons expressing cFos was then calculated. The E/I balance was defined as the ratio between the fraction of cFos-expressing pyramidal neurons and either c-Fos-expressing ErbB4-positive neurons or cFos-expressing parvalbumin-positive interneurons.

### Intracerebroventricular NRG1 injection procedures

Mice were anesthetized with urethane (1.25 g kg^−1^; i.p.) or isoflurane (2% Wako Pure Chemical Industries, Tokyo, Japan) and placed in a stereotaxic frame. In electrophysiological experiments, stainless-steel cannulae (Plastics One, Roanoke, VA, USA) were implanted stereotaxically into the lateral ventricle (0.22 mm posterior to the bregma, 1 mm lateral to the midline and 2 mm ventral to the dural surface). To analyze the effect of NRG1_177-246_ infusion on the seizure score and cFos expression after KA administration, stainless-steel guide cannulae (Plastics One) were placed above the lateral ventricle and secured in the skull using dental cement. Dummy cannulae (Plastics One) were then inserted into the guide cannulae and replaced 1 week later with the injection cannulae, which projected 0.5 mm below the tip of the guide cannulae. Recombinant human epidermal growth factor-like domain of NRG1 (5 μm in PBS; NRG1_177-246_; PeproTech, Rocky Hill, NJ, USA) or vehicle was delivered either 10 min or 3 h after KA administration via a Hamilton syringe mounted on an infusion pump (CFV-2100; Nihon Koden, Tokyo, Japan) at a flow rate of 0.2 μl min^−1^ for 10 min. Control infusions used only PBS.

### Statistical analysis

All data are expressed as the mean and standard error of the mean (SEM). Statistical significance was determined as indicated by applying a two-tailed unpaired Student's *t*-test (normal distribution), the Mann–Whitney *U* test (non-normal distribution), one-way ANOVA with Tukey's or Dunnett's *post-hoc* test, or two-way ANOVA with the Sidak *post-hoc* test (comparison of multiple groups). No statistical methods were used to predetermine sample sizes, but the sample sizes used are similar to those reported in previous publications.^18, 36^ The criterion for statistical significance was *P*<0.05.

## Results

### Neuropsin is activated during KA-evoked seizures

**Figure 1:**
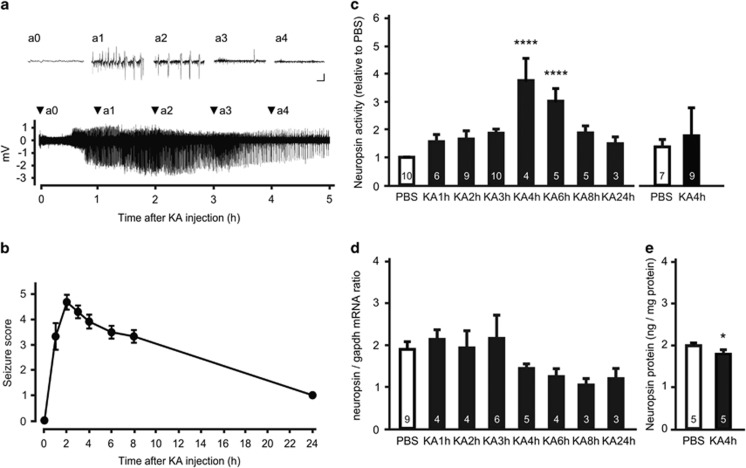
Seizures activate hippocampal neuropsin *in vivo.* (**a**) Representative trace of a local field potential in the hippocampal CA1 region after intraperitoneal injection of kainate (kainic acid, KA). Insets (a0–a4) show the waveforms at the times indicated by arrowheads. Scale bars, 1 mV and 1 s. (**b**) Time course for the change in seizure scores after KA administration. The seizure score increased sharply within 1 h after KA administration and remained high for at least 8 h (*n*=13 mice). (**c**) Endogenous neuropsin activity at various time points in the hippocampus of mice in which status epilepticus was induced (left) or not induced (right) by KA administration. Neuropsin activity was significantly higher 4 and 6 h after KA administration only in those mice with induced status epilepticus (F_(7,44)_=7.902; ****P<0.0001, one-way ANOVA with Dunnett's *post-hoc* test). (**d**) Quantitative real-time PCR analysis of neuropsin mRNA in mouse hippocampus after KA administration. Data are shown as neuropsin mRNA relative to *Gapdh* mRNA. The expression of neuropsin mRNA was not affected by seizure activity (one-way ANOVA, F_(7,30)_=1.597, *P*=0.1746). (**e**) Concentration of neuropsin protein in the mouse hippocampus 4 h after PBS (white bars) or KA (black bars) administration. The amount of neuropsin protein fell slightly at 4 h after KA administration (*t*-test, *t*_8_=2.471; *P=0.0386 vs PBS-treated mice). Error bars indicate the SEM. Numbers inside columns indicate *n*. PBS, phosphate buffered saline; SEM, standard error of the mean.

To investigate whether hippocampal neuropsin activity is affected by seizures, we used an animal model of temporal lobe epilepsy. A microelectrode was inserted into the CA1 pyramidal cell layer of C57BL/6 J mice to measure neural activity, and behavioral seizures were induced by i.p. injection of KA. KA administration induced an epileptic discharge accompanied by generalized tonic-clonic behavioral seizures (Figures 1a and b). Although spiking rates and burst amplitudes peaked at about 2 h and 2.5 h, respectively, neuropsin activity peaked at 4 h (one-way ANOVA, F_(7,44)_=7.902, *P*<0.0001 vs PBS-treated mice; Figure 1c, left). Neuropsin activity remained significantly elevated at 6 h (one-way ANOVA, *P*<0.0001 vs PBS-treated mice) before finally returning to control levels (Figure 1c, left). No change in neuropsin activity was detected in mice that did not develop status epilepticus after several injections of KA, (*t*-test, *t*_14_=0.3421, *P*=0.7373; Figure 1c, right). Neuropsin mRNA expression remained unchanged or decreased slightly (but not significantly) both during and after seizures (one-way ANOVA, F_(7,30)_=1.597, *P*=0.1746; Figure 1d), and the amount of neuropsin protein in the hippocampus of mice injected with KA (1.825±0.075 ng mg^−1^ protein) was slightly, but significantly, lower than that of controls (PBS: 2.028±0.032 ng mg^−1^ protein; *t*-test, *t*_8_=2.471, *P*=0.0386; Figure 1e). These results indicate that the proteolytic activity of neuropsin is enhanced by KA-induced seizures without any increase in transcription and translation; a finding consistent with that of a previous study.^22^

### Neuropsin contributes to KA-induced status epilepticus

**Figure 2:**
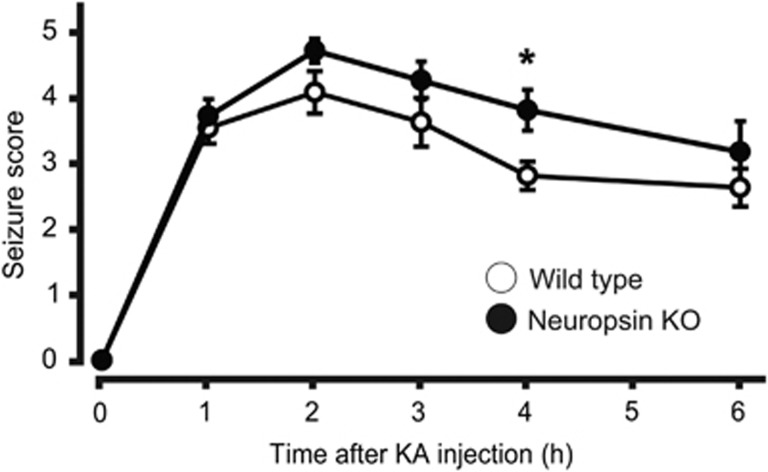
Time course for the seizure score in neuropsin-knockout (KO) mice administered kainate (KA). Mice received repeated injections of KA. KA-treated neuropsin-KO mice (black circles; *n*=11 mice) showed progression of seizures compared with KA-treated wild-type mice (white circles; *n*=11 mice, two-way ANOVA, F_(1,20)_=5.744; *P=0.0264). Seizure score of neuropsin-KO mice was significantly higher than that of wild-type mice only at the 4 h after KA administration (*P=0.046; two-way ANOVA with the Sidak *post-hoc* test). Error bars indicate standard error of the mean.

Because neuropsin activity was upregulated 4 and 6 h after KA injection, we examined whether neuropsin contributes to KA-induced status epilepticus using neuropsin-KO mice at these time points. Although we observed no significant differences in the total KA dose required to obtain a continuous motor seizure (*t*-test, *t*_20_=0.3043, *P*=0.7641) and onset of status epilepticus (*t*-test, *t*_20_=0.1743, *P*=0.8634) between wild-type and neuropsin-KO mice (Supplementary Table 1), the severity of the seizures in neuropsin-KO mice after KA injection was significantly greater than that in wild-type mice only at the 4 h time point (*n*=11 mice, two-way ANOVA, F_(1,20)_=5.744, *P*=0.046; Figure 2). Neuropsin-KO mice tended to exhibited stage 4 or 5 behavior for longer than wild-type mice, although the difference was not statistically significant (neuropsin-KO, 110.9±7.9 min, *n*=11; wild-type, 83.1±12.4 min, *n*=11; *t*-test, *t*_20_=1.886, *P*=0.074). These data indicate that deleting neuropsin leads to progression of KA-induced status epilepticus.

### Neuropsin liberates the ligand moiety of mNRG1 from heparan sulfate proteoglycans

**Figure 3:**
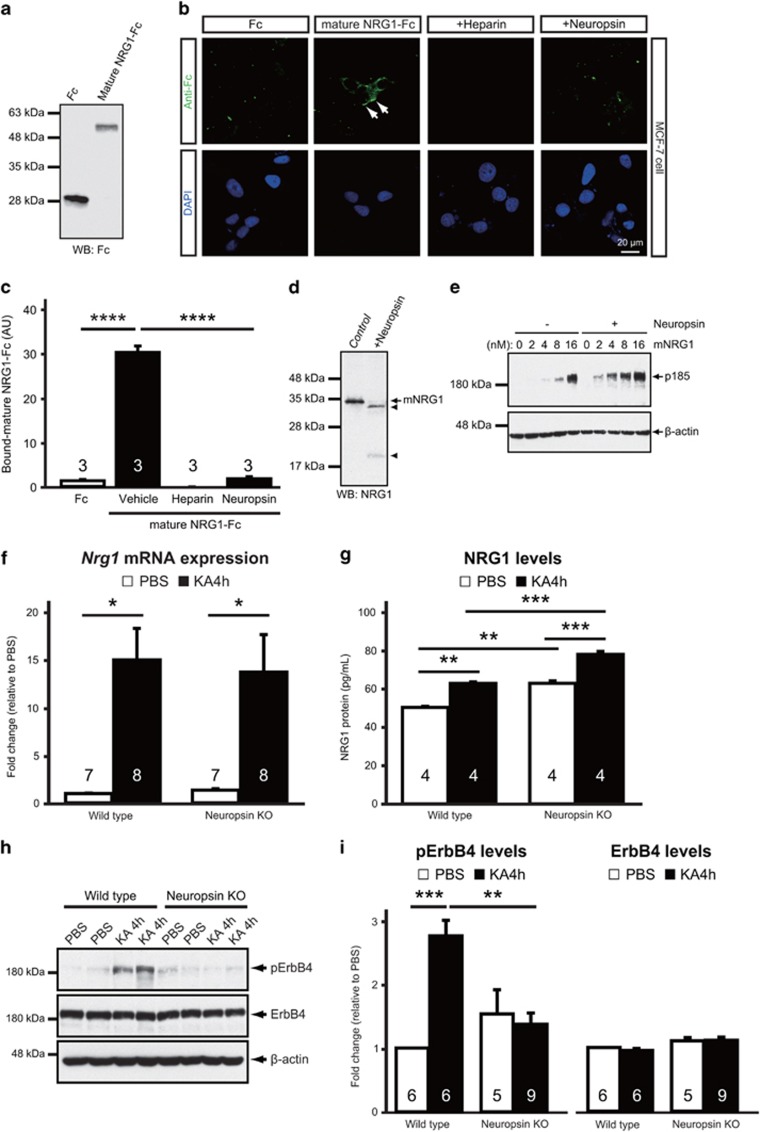
Neuropsin modulates NRG1–ErbB4 signaling. (**a**) Western blots with control Fc protein and mouse mNRG1-Fc protein probed with an anti-Fc antibody. (**b**) MCF-7 cells were treated with mNRG1-Fc with or without heparin or neuropsin. Fc and mNRG1-Fc were detected with Alexa Fluor 488-conjugated anti-rabbit IgG (green, upper panels). The nuclei were labeled with DAPI (blue, lower panels). mNRG1-Fc binds to MCF-7 cells, and this binding is abolished by treatment of the cells with heparin or neuropsin. (**a, b**) Each show one representative result from three independent experiments. (**c**) Quantitative analysis of Fc (white bar) or mNRG1-Fc (black bars) immunofluorescent staining in MCF-7 cells after treatment with vehicle, heparin or neuropsin (30–54 cells; *n*=3 independent experiments) as shown in **b**. Heparin or neuropsin administration significantly reduced the immunoreactivity of mNRG1-Fc (one-way ANOVA, F_(3,8)_=247.0; ****P<0.0001 vs vehicle). Means (AU, arbitrary units) and the SEM are shown. ****P<0.0001; one-way ANOVA with Tukey's *post-hoc* test. (**d**) Recombinant human mNRG1 was preincubated without (Control) or with neuropsin (+Neuropsin) and then subjected to western blotting with an anti-C-terminal mNRG1 antibody. Proteolytic cleavage of mNRG1 by neuropsin results in the appearance of two major immunoreactive fragments (arrowheads), corresponding to cleaved mNRG1 C-terminal fragments. Similar results were seen in three independent experiments. (**e**) MCF-7 cells were treated with human mNRG1 at the indicated concentrations with or without neuropsin. Phosphorylation of p185 (ErbB), the receptor for mNRG1, was analyzed by western blotting with an anti-phosphotyrosine antibody. The β-actin control blot verified equivalent loading. Similar results were obtained in three independent experiments. (**f**) *Nrg1 type I* mRNA levels are upregulated in the hippocampus of both wild-type (*P=0.0126 vs PBS) and neuropsin-KO (*P=0.0312 vs PBS) mice 4 h after KA administration (black bars) compared with those following PBS injection (white bars) (one-way ANOVA, F_(3,26)_=6.654; *P*=0.0017). *Nrg1 type I* mRNA was quantified relative to the expression of *Gapdh* mRNA using real-time PCR. The fold change was normalized to PBS in wild-type mice. (**g**) Concentration of NRG1 protein, measured using ELISA, in hippocampal homogenates from wild-type or neuropsin-KO mice 4 h after PBS (white bars) or KA (black bars) administration. KA-induced upregulation of NRG1 protein levels in neuropsin-KO mice is greater than that in wild-type mice (one-way ANOVA, F_(3,12)_=39.14; ***P=0.0004 for the wild-type after KA administration vs neuropsin-KO after KA administration). (**h**) Representative western blots showing the levels of phosphorylated ErbB4 (pErbB4), total ErbB4 and β-actin in the hippocampus from wild-type or neuropsin-KO mice 4 h after PBS or KA administration. (**i**) Quantitative densitometric analysis of western blots. Ratios for pErbB4/ErbB4 (left) and ErbB4/β-actin (right) are shown. The fold change was normalized to PBS in wild-type mice. pErbB4 protein levels are upregulated by seizure activity in wild-type mice (one-way ANOVA, F_(3,22)_=9.601, ***P=0.0003 vs PBS) but not in neuropsin-KO mice (*P*=0.9637 vs PBS). β-actin served as the loading control. Error bars indicate the SEM. *P<0.05, **P<0.01, ***P<0.001 and ****P<0.0001; one-way ANOVA with Turkey's *post-hoc* test. Numbers inside columns indicate *n*. KA, kainate; KO, knockout; NRG1, neuregulin 1; PBS, phosphate buffered saline; SEM, standard error of the mean.

To analyze the role of neuropsin during proteolysis of natural substrates, we examined the processing of mNRG1, the native substrate of neuropsin,^26^ in MCF-7 cells (which express abundant amounts of ErbB receptors, including ErbB4).^37^ We first generated a recombinant chimeric protein, called mouse mNRG1*-*Fc, comprising the extracellular domain of mouse NRG1 fused to the Fc portion of rabbit Ig (Figure 3a) and bath-applied it to MCF-7 cells. Strong immunoreactive signals for mouse mNRG1*-*Fc, but not the Fc control protein, were observed on MCF-7 cells (one-way ANOVA, *P*<0.0001 vs Fc; Figures 3b and c), indicating cell-surface binding. Co-incubation in the presence of the heparan sulfate analog, heparin or neuropsin reduced the mouse mNRG1*-*Fc immunoreactivity (one-way ANOVA, *P*<0.0001 vs vehicle; Figures 3b and c), suggesting that mNRG1 was released from heparan sulfate proteoglycans (HSPGs) by neuropsin-mediated proteolytic cleavage. As HSPG-bound mNRG1 does not induce phosphorylation of its receptor (ErbB) in cells,^38^ we hypothesized that it must be processed by neuropsin for this effect to take place. To test this, we investigated the tyrosine phosphorylation status of ErbB in MCF-7 cells incubated with different concentrations of recombinant human mNRG1 in the presence or absence of neuropsin. As shown in Figures 3d and e, neuropsin cleaved human mNRG1 at multiple sites to yield two major bands corresponding to the cleaved mNRG1 C-terminal fragments shown previously;^26^ it also increased phosphorylation of ErbB in the presence of very low concentrations of human mNRG1 (2*–*16 nm). At high concentrations (16 nm), a small proportion of mNRG1 was not inhibited by HSPGs, even in the absence of neuropsin, presumably due to saturation of mNRG1 binding sites on HSPGs. Taken together, these results suggest that neuropsin activates and enhances NRG1 signaling.

### NRG1–ErbB4 signaling is actuated by neuropsin in the epileptic hippocampus

Because cleavage by neuropsin and the subsequent release of the ligand moiety of NRG1 are necessary for the activation of ErbB4, we hypothesized that neuropsin, when activated by epileptic seizures, stimulates NRG1–ErbB4 signaling *in vivo*. Four to six hours after KA administration, when neuropsin was highly activated (Figure 1c), *Nrg1 type I* mRNA expression and ErbB4 phosphorylation levels were simultaneously increased in mouse hippocampi (Supplementary Figure 2). Hence, we examined whether neuropsin-KO mice exhibit abnormal NRG1–ErbB4 signaling during seizures. At 4 h post-KA injection, *Nrg1 type I* mRNA expression in neuropsin-KO mice increased to the same extent as that in wild-type mice (one-way ANOVA, *P*=0.9889; Figure 3f); however, ELISA revealed that ‘basal' levels of NRG1 (in PBS-injected animals) were higher in KO than in wild-type mice (one-way ANOVA, *P*=0.0017 vs PBS-injected wild-type mice) and that the amount of NRG1 protein after KA administration was also greater in KO than in wild-type mice (one-way ANOVA, *P*=0.0004 vs KA-injected wild-type mice; Figure 3g), indicating that much of the NRG1 protein in neuropsin-KO mice remains bound to HSPGs. Moreover, western blotting showed that phosphorylated ErbB4 protein levels in the hippocampi of wild-type mice at 4 h after KA injection increased significantly (one-way ANOVA, F_(3,22)_=9.601, *P*=0.0003 vs PBS); this was not the case in neuropsin-KO mice (one-way ANOVA, *P*=0.9637 vs PBS; Figures 3h and i). On the other hand, the total amount of ErbB4 protein did not change significantly in either group, even after KA administration (one-way ANOVA, F_(3,22)_=2.855, *P*=0.0604; Figures 3h and i). Taken together, these data suggest that processing of NRG1 by neuropsin is critical for induction of NRG1–ErbB4 signaling *in vivo*.

### Significant decrease of seizure-related cFos expression in parvalbumin-expressing interneurons of neuropsin-KO mice

Because NRG1 is known to regulate the excitability of parvalbumin-expressing interneurons expressing ErbB4,^39^ it was important to examine whether neuropsin also contributed to GABAergic cellular activity *in vivo*. Therefore, we assessed the activation of ErbB4-expressing neurons in the hippocampal CA1 region after KA administration using cFos immunoreactivity as a marker for neuronal activity. Although no cFos expression was detected in the CA1 region of PBS-injected control mice, an abundance of cFos-labeled pyramidal neurons was observed at 1 h post-KA administration (249.6±14.4 neurons per area; Supplementary Figure 2); however, a few ErbB4-positive neurons showed very slight cFos immunoreactivity (1.9±0.6 neurons per area; Supplementary Figure 3). At 4 h after KA administration, the number of cFos-labeled pyramidal neurons decreased significantly (30.0±3.4 neurons per area; Mann–Whitney *U* test, *P*=0.0286 vs 1 h after KA administration; Supplementary Figure 3) and in parallel with a reduction in local field potential activity (Figure 1a). Nevertheless, strong cFos immunoreactivity was observed in most ErbB4-labeled neurons (17.6±1.7 neurons per area; Mann–Whitney *U* test, *P*=0.0286 vs 1 h after KA administration; Supplementary Figures 3 and 4). ErbB4-labeled neurons exhibiting strong cFos immunoreactivity were positive for parvalbumin, but not for calcium/calmodulin-dependent protein kinase (CAMKII), a marker of excitatory neurons (Supplementary Figure 4 and Supplementary Table 2). Consistent with the timing of cFos activation in ErbB4-labeled neurons, the number of parvalbumin-positive interneurons expressing cFos was higher at 4 h than at 1 h after KA administration (1 h after KA administration, 1.7±0.5 neurons per area; 4 h after KA administration, 11.9±1.4 neurons per area; Mann–Whitney *U* test, *P*=0.0286; Supplementary Figure 5). These results show that the cFos expression after the onset of seizures occurs later in ErbB4-expressing parvalbumin-positive interneurons (4 h) than it does in pyramidal neurons (1 h), and that the timing of expression in parvalbumin-positive interneurons corresponds with the peak of activation of neuropsin after KA injection.

**Figure 4:**
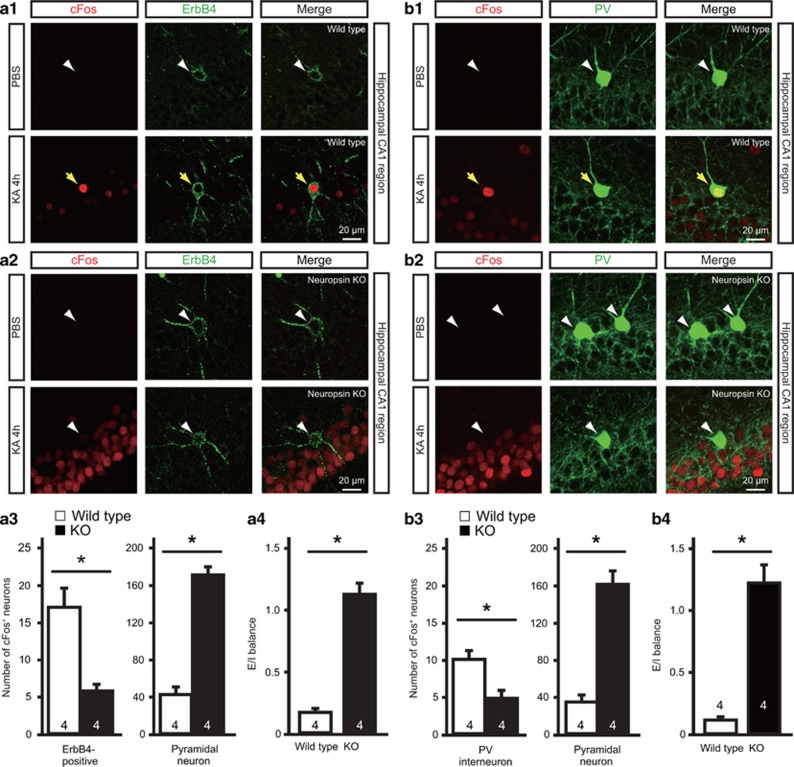
Neuropsin-KO mice show reduced cFos expression in ErbB4-positive neurons and inhibitory activity and enhanced excitatory activity after seizure. (**a**1, **a**2) Expression of cFos in ErbB4-positive and pyramidal neurons of wild-type (**a**1) or neuropsin-KO (**a**2) mice 4 h after PBS (upper panel) or KA (lower panel) administration. Fluorescent labeling of the hippocampal CA1 region shows strong cFos immunoreactivity (red) in ErbB4-positive neurons (green) in wild-type mice, but only faint staining in neuropsin-KO mice. Yellow arrows indicate ErbB4-positive neurons expressing cFos. White arrowheads indicate ErbB4-positive neurons that do not express cFos. (**a**3) Quantitative analysis of cFos immunofluorescent labeling of ErbB4-positive and pyramidal neurons of the wild-type (white bars) and neuropsin-KO (black bars) mice shown in **a**1 and **a**2. The number of cFos-expressing ErbB4-positive neurons in neuropsin-KO mice was significantly lower than that in wild-type mice (Mann–Whitney *U* test, *P=0.0286 vs wild-type); by contrast, the number of cFos-immunoreactive pyramidal neurons from neuropsin-KO mice was significantly higher than that in wild-type mice (Mann–Whitney *U* test, *P=0.0286 vs wild-type). Data are expressed as the mean and SEM. (**a**4) E/I balance, defined as the ratio between the fractions of pyramidal neurons and ErbB4-positive neurons expressing cFos, in wild-type (white bar) and neuropsin-KO (black bar) mice. The E/I balance in neuropsin-KO mice was significantly higher than that in wild-type mice (Mann–Whitney *U* test, *P=0.0286 vs wild-type). (**b**1, **b**2) Expression of cFos in parvalbumin (PV)-positive interneurons (arrows) and pyramidal neurons in wild-type (**b**1) or neuropsin-KO (**b**2) mice 4 h after PBS (upper panel) or KA (lower panel) administration. Fluorescent labeling of the hippocampal CA1 region shows strong cFos immunoreactivity (red) in parvalbumin-positive interneurons (green) in wild-type mice, but only faint staining in neuropsin-KO mice. Yellow arrows indicate parvalbumin-positive neurons expressing cFos. White arrowheads indicate parvalbumin-positive neurons that do not express cFos. (**b**3) Quantitative analysis of cFos immunofluorescent labeling in PV-positive interneurons and pyramidal neurons of wild-type (white bars) and neuropsin-KO (black bars) in the mice shown in **b**1 and **b**2. The number of cFos-expressing parvalbumin-positive interneurons in neuropsin-KO mice was significantly lower than that in wild-type mice (Mann–Whitney *U* test, *P=0.0286 vs wild-type); by contrast, the number of cFos-immunoreactive pyramidal neurons in neuropsin-KO mice was significantly higher than that in wild-type mice (Mann–Whitney *U* test, *P=0.0286 vs wild-type). Data are expressed as the mean and SEM. (**b**4) E/I balance, defined as the ratio between the fractions of pyramidal neurons and parvalbumin-positive interneurons expressing cFos in wild-type (white bar) and neuropsin-KO (black bar) mice. The E/I balance in neuropsin-KO mice was significantly higher than that in wild-type mice (Mann–Whitney *U* test, *P=0.0286 vs wild-type). Numbers inside columns indicate *n*. KA, kainate; KO, knockout; PBS, phosphate buffered saline; SEM, standard error of the mean.

**Figure 5:**
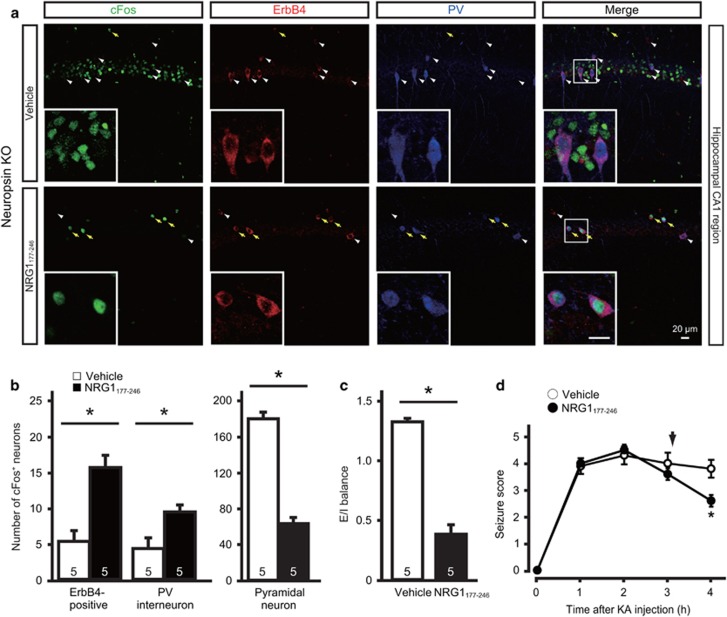
Effect of intracerebroventricular injection of NRG1_177-246_ on E/I balance and KA-induced seizure in neuropsin-KO mice. (**a**) Expression of cFos (green) in ErbB4-positive neurons (red), parvalbumin (PV)-positive interneurons (blue) and pyramidal neurons at 4 h after KA administration in vehicle- (upper panel) or NRG1_177-246_-injected (lower panel) neuropsin-KO mice. Yellow arrows indicate ErbB4-positive neurons expressing cFos. White arrowheads indicate ErbB4-positive neurons that do not express cFos. Regions inside the boxes are enlarged in the inset images (scale bar, 20 μm). (**b**) Quantitative analysis of cFos expression in ErbB4-positive neurons, PV-positive interneurons, and pyramidal neurons in the vehicle- (white bars) and NRG1_177-246_-injected (black bars) neuropsin-KO mice shown in **a**. Significantly more ErbB4-positive neurons and parvalbumin-positive interneurons were immunoreactive for cFos in NRG1_177-246_-injected neuropsin-KO mice than in vehicle-injected mice (Mann–Whitney *U* test, *P=0.0286 vs vehicle); by contrast, the number of cFos-immunoreactive pyramidal neurons in NRG1_177-246_-injected neuropsin-KO mice was significantly lower than that in vehicle-injected mice (Mann–Whitney *U* test, *P=0.0286 vs vehicle). (**c**) E/I balance, defined as the ratio between the fractions of pyramidal neurons and parvalbumin-positive interneurons expressing cFos, in vehicle- (white bar) and NRG1_177-246_-injected (black bar) neuropsin-KO mice. The E/I balance in NRG1_177-246_-injected neuropsin-KO mice was significantly lower than that in vehicle-injected mice (Mann–Whitney *U* test, *P=0.0286 vs vehicle). Numbers inside the columns indicate *n*. (**d**) Time course of the seizure score in vehicle- or NRG1_177-246_-injected neuropsin-KO mice after KA administration. The seizure score for NRG1_177-246_-injected mice (black circles; *n*=5 mice) was significantly lower than that for vehicle-injected mice (white circles; *n*=5 mice) only at 4 h after KA administration (F_(1,8)_=1.134; *P=0.019; two-way ANOVA with the Sidak *post-hoc* test). The arrow denotes the point of vehicle or NRG1_177-246_ injection. Error bars indicate the SEM. E/I balance, excitation-inhibition balance; KA, kainate; KO, knockout; NRG1, neuregulin 1.

To determine whether the appearance of cFos labeling in ErbB4-positive neurons was mediated by neuropsin, we compared hippocampal tissues from neuropsin-KO and wild-type mice 4 h after KA administration (Supplementary Figure 6 and Figure 4a1–a3). The number of cFos-expressing ErbB4-labeled neurons in neuropsin-KO mice (5.8±0.9 neurons per area) was significantly lower than that in wild-type mice (17.0±2.6 neurons per area; Mann–Whitney *U* test, *P*=0.0286; Figure 4a3, left panel); however, the number of cFos-positive pyramidal neurons in neuropsin-KO mice (170.9±8.6 neurons per area) was significantly higher than that in wild-type mice (42.1±8.1 neurons per area; Mann–Whitney *U* test, *P*=0.0286; Figure 4a3, right panel). The fraction of cFos-expressing pyramidal neurons represented 11.8±2.5% (wild-type) and 42.9±4.5% (neuropsin-KO) of the total number of pyramidal neurons, whereas the fraction of cFos-expressing ErbB4-positive neurons represented 72.2±4.4% (wild-type) and 39±4.0% (neuropsin-KO) of the total number of ErbB4-positive neurons. We defined the ratio between the fractions of cFos-labeled pyramidal neurons and cFos-labeled ErbB4-positive neurons as the E/I balance. The E/I balance was elevated in neuropsin-KO mice compared with the wild-type mice (Mann–Whitney *U* test, *P*=0.0286; Figure 4a4). However, the density of ErbB4-positive neurons was not significantly different between the groups (ErbB4-positive neuron numbers per 500 000 μm^3^ in the CA1 region were 20±3.6 (wild-type) and 19.5±3.3 (neuropsin-KO); *n*=4 mice each, Mann–Whitney *U* test, *P*=0.8571). These results suggest that neuropsin deficiency impairs the activation of ErbB4-positive neurons, resulting in hyperactivity of pyramidal neurons in the stimulated state and breakdown of the E/I balance. Similarly, compared with that in wild-type mice, only half the number of cFos-expressing parvalbumin-positive interneurons (wild-type, 10±1.2 neurons per area; neuropsin-KO, 4.8±1.1 neurons per area; Mann–Whitney *U* test, *P*=0.0286; Supplementary Figure 6 and Figure 4b1–b3) and a higher level of E/I balance, defined as the ratio between the fraction of cFos-labeled pyramidal neurons and cFos-labeled parvalbumin-positive interneurons (Mann–Whitney *U* test, *P*=0.0286; Figure 4b4), were detected in neuropsin-KO mice. The fraction of cFos-expressing parvalbumin-positive neurons represented 78.8±7.2% (wild-type) and 38.3±3.8% (neuropsin-KO) of the total number of parvalbumin-positive neurons. The altered E/I balance and lower cFos expression by inhibitory neurons in neuropsin-KO mice were reversed by intracerebroventricular injection of recombinant NRG1_177-246_ at 3 h after KA administration (Figures 5a–c). Interestingly, injection of NRG1_177-246_ significantly reduced seizure severity at 4 h after KA administration in these mice (two-way ANOVA, F_(1,8)_=1.134, *P*=0.0186; Figure 5d). Taken together, these results indicate that seizure activity activates ErbB4-expressing parvalbumin-positive interneurons via neuropsin–NRG1 signaling.

### Hippocampal gamma oscillations are impaired in neuropsin-KO mice

**Figure 6:**
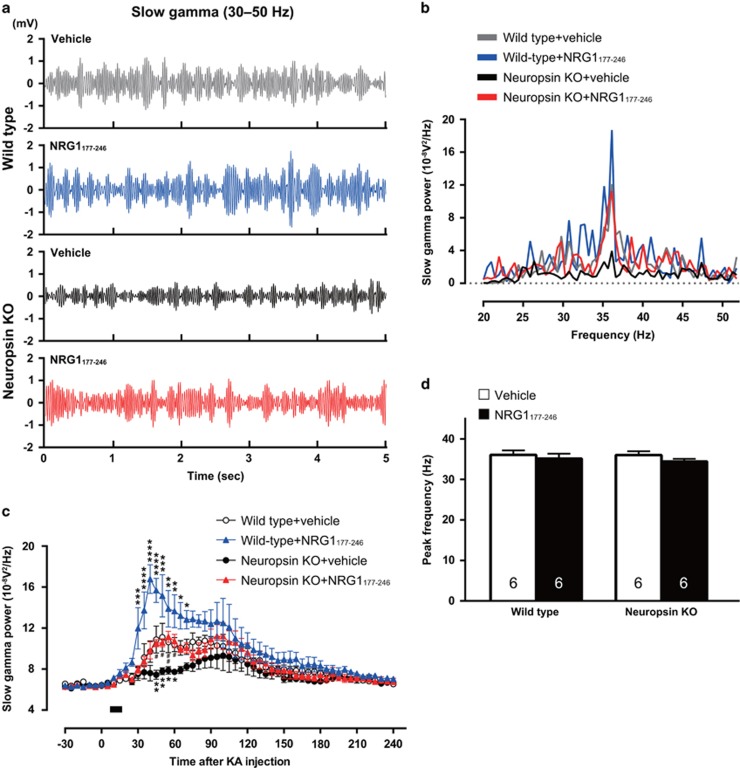
Disrupted slow gamma oscillations in neuropsin-KO mice. (**a, b**) Representative local field potential traces (30–50 Hz) (**a**) and power spectra (**b**) of KA-induced slow gamma oscillations in wild-type mice after vehicle (gray) or NRG1_177-246_ injection (blue) and in neuropsin-KO mice after vehicle (black) or NRG1_177-246_ injection (red). (**c**) Time course for the mean power (and SEM) (30–50 Hz) of KA-induced slow gamma oscillations in wild-type mice after vehicle (white circles; *n*=6 mice) or NRG1_177-246_ injection (blue triangles; *n*=6 mice) and in neuropsin-KO mice after vehicle (black circles; *n*=6 mice) or NRG1_177-246_ injection (red triangles; *n*=6 mice). The power of the slow gamma oscillations in the CA1 region in neuropsin-KO mice was less than that in wild-type mice, but recovered after injection of NRG1_177-246_ (F_(3,20)_=6.343, *P*=0.0034; two-way ANOVA). Significant differences between wild-type and neuropsin-KO mice were observed at 45–60 min after vehicle injection (45 min, **P=0.0044; 50 min, **P=0.0067; 55 min, *P=0.0347; 60 min, *P=0.0459; two-way ANOVA with the Sidak *post-hoc* test). Significant differences between wild-type mice were seen from 30 to 70 min after injection of vehicle or NRG1_177-246_ (30 min, ***P=0.0002; 35 min, ****P<0.0001; 40 min, ****P<0.0001; 45 min, ****P<0.0001; 50 min, ***P=0.0005; 55 min, **P=0.0082; 60 min, **P=0.0082; 65 min, *P=0.0117; 70 min, *P=0.0462; two-way ANOVA with the Sidak *post-hoc* test). There were significant differences between neuropsin-KO mice at 45–60 min post-injection with vehicle or NRG1_177-246_ (45 min, ^#^*P*=0.0145; 50 min, ^#^*P*=0.0329; 55 min, ^##^*P*=0.0075; 60 min, ^#^*P*=0.0183; two-way ANOVA with the Sidak *post-hoc* test). NRG1_177-246_ or vehicle was injected during the time period indicated by the bar. (**d**) Bar graph showing the mean (and SEM) peak frequency of KA-induced slow gamma oscillations in wild-type mice and neuropsin-KO mice after injection of vehicle (white bar) or NRG1_177-246_ (black bar). No difference in the peak frequency of slow gamma oscillations was detected between the groups (F_(3,20)_=0.6128, *P*=0.6146; one-way ANOVA with Tukey's *post-hoc* test). Numbers inside columns indicate *n*. KA, kainate; KO, knockout; NRG1, neuregulin 1; SEM, standard error of the mean.

The activity of parvalbumin-positive interneurons is critical for driving gamma oscillations,^3, 4, 7, 40^ and NRG1 increases the power of gamma oscillations via activation of ErbB4.^41^ Because we found that neuropsin contributed to the activation of ErbB4-expressing parvalbumin-positive interneurons, we investigated whether neuropsin is involved in hippocampal gamma activity. The slow (30–45 Hz) and fast (65–120 Hz) gamma oscillations in the local field potential were recorded in the hippocampal CA1 region of anesthetized mice. Following a 30 min baseline recording the mice received a single injection of KA (25 mg kg^−1^, i.p.). The power level of slow gamma oscillations in neuropsin-KO mice during baseline recording was similar to that in wild-type mice (5–30 min before KA administration: wild-type, 6.486±0.089 10^−8^V^2^/Hz; neuropsin-KO, 6.344±0.085 10^−^^8^V^2^/Hz; one-way ANOVA, F_(3,20)_=0.8335, *P*=0.5739); however, it was significantly lower after KA administration (two-way ANOVA, F_(3,20)_=6.343, *P*=0.0034; Figures 6a–c). Lateral cerebroventricular injection of recombinant NRG1_177-246_ into neuropsin-KO mice led to complete recovery of the power of slow gamma oscillations to wild-type levels (*P*<0.05 at 45, 50 and 60 min, and *P*<0.01 at 55 min after KA administration between KO+vehicle and KO+NRG1_177-246_; no significant difference between wild-type+vehicle and KO+NRG1_177-246_; Figures 6a–c). NRG1_177-246_ also increased the power of these oscillations in wild-type mice (Figures 6a–c). The peak frequency was the same for all groups (one-way ANOVA, F_(3,20)_=0.6128, *P*=0.6146; Figure 6d). Both wild-type and neuropsin-KO mice exhibited fast gamma oscillation of similar power and peak frequency both before and after KA administration (two-way ANOVA, F_(3,20)_=1.834, *P*=0.1735; Supplementary Figure 7). Lateral cerebroventricular injection of NRG1_177-246_ increased the power to the same level in both groups of mice (Supplementary Figure 7). Thus, neuropsin-KO mice showed a clear deficit in slow, but not fast, gamma oscillation activity, which was ameliorated by addition of NRG1 comprising the ligand domain for ErbB4.

## Discussion

The main findings of the present study are that neuropsin is involved in the generation of slow gamma oscillations and in the delayed seizure-related activation of ErbB4-expressing parvalbumin-positive interneurons, which regulate the hippocampal E/I balance *in vivo*. The E/I balance in neuropsin-KO mice, in which NRG1–ErbB4 signaling was considerably impaired, was also disturbed and accompanied by hypoactivity of parvalbumin-expressing interneurons and pyramidal neuron hyperexcitability. The power of the slow gamma oscillations was reduced, whereas their peak frequency was unchanged. The E/I imbalance and reduced gamma power were restored by application of NRG1_177–246_, a synthetic functional peptide derived from NRG1 but lacking the heparin-binding domain. These results suggest that the neuropsin–NRG1 signaling system is essential to maintain the balance of network excitability and the magnitude of slow gamma oscillations.

Single parvalbumin-positive GABAergic interneurons innervate about a thousand pyramidal neurons.^42^ This central inhibitory control on pyramidal neurons is thought to produce rhythmic oscillatory activity,^3, 4^ and to contribute to brain cognition and memory formation.^43, 44^ Although accumulating evidence suggests that E/I networks are engaged in the processing and storage of information,^1^ the mechanism of activity-dependent regulation of GABAergic interneurons, of which most are parvalbumin-positive, is not well understood. A previous study reported that the expression of cFos in excitatory neurons precedes its expression in inhibitory interneurons after the onset of behavioral seizures in a mouse model of epilepsy.^45^ Consistently, we observed that parvalbumin-positive GABAergic interneurons exhibited strong cFos immunoreactivity 4 h after KA administration, but only faint immunoreactivity at earlier time points. By contrast, neuropsin-KO mice exhibited a larger number of cFos-expressing pyramidal neurons and fewer cFos-expressing parvalbumin-positive interneurons than wild-type mice, suggesting that cFos expression in parvalbumin-positive interneurons depends on neural activity-dependent activation of neuropsin. We also found that neuropsin–NRG1–ErbB4 signaling was enhanced in parallel with cFos expression in parvalbumin-positive interneurons. Phosphorylation of ErbB4, induced by NRG1 binding, increases the excitability of parvalbumin-positive interneurons, but not pyramidal neurons, by regulating Kv1.1, a voltage-gated potassium channel on parvalbumin-positive interneurons;^39, 46^ it also drives GABA release from parvalbumin-expressing interneurons.^47^ Therefore, parvalbumin-expressing interneuron activity may be regulated by neuropsin–NRG1 signaling at the excitatory synapse. This hypothesis is supported by our electrophysiological experiments which showed impaired GABAergic transmission in neuropsin-KO mice and its rescue by application of NRG1_177-246_. Therefore, neuropsin–NRG1–ErbB4 signaling may be one of the molecular mechanisms activating parvalbumin-expressing interneurons during seizures, although it remains to be determined whether these two events are causally related.

Gamma oscillations are thought to be involved in higher cognitive processes, such as working memory, and to be associated with phase-locked rhythmic activity of basket cells expressing parvalbumin.^48^ Indeed, a recent optogenetic study showed that rhythmic stimulation of parvalbumin-expressing interneurons increases oscillation activity at the gamma frequency, but not at other frequencies.^3^ Previous studies reported that NRG1 increases gamma oscillation power in the hippocampus and prefrontal cortex through activation of ErbB4 on parvalbumin-expressing interneurons, though the power is reduced in *Erbb4*-null mutant slices without any defect in frequency;^41, 49^ our present study is consistent with these observations. Overall, these results implicate NRG1–ErbB4 signaling in the molecular mechanisms that underlie the magnitude of gamma oscillations. However, they do not rule out the possibility that NRG1 regulates gamma frequency, because hippocampal slices from transgenic mice overexpressing NRG1 exhibited a reduced frequency of carbachol-induced gamma oscillations.^50^ In the present study, neuropsin-KO mice had reduced gamma power but normal peak frequency; injection of NRG1_177-246_ restored the power impairment to wild-type levels and was accompanied by a slight, but statistically insignificant decrease in peak frequency. These results indicate that neuropsin modulates gamma power through NRG1 signaling, although the actuation of NRG1 signaling by an additional system other than neuropsin proteolysis cannot be excluded. Gamma oscillations correspond to synchronous inhibitory post-synaptic potentials arising from fast-spiking interneurons.^48^ Consistent with this finding, NRG1 enhances the synchrony between firing pairs of fast-spiking interneurons and pyramidal neurons by activating ErbB4.^49^ Hence, ablation of neuropsin–NRG1 signaling may impair the synchronous activity of neuronal assemblies, contributing to cognitive impairment in several psychiatric diseases such as schizophrenia.^51^ As such, the impairment of spatial working memory observed in neuropsin-KO mice has been proposed to be due to a dysfunction of gamma oscillations mediated by parvalbumin-expressing interneurons;^15^ this idea is supported by the finding that functional removal of parvalbumin-expressing interneurons impairs spatial working memory.^52^

We found that neuropsin-KO mice exhibit pyramidal neuron hyperexcitability due to reduced parvalbumin-expressing interneuron activity, although it is not clear whether the effects of neuropsin on cFos labeling reflect changes in synaptic and neuronal activity. Disturbance of the E/I balance underlies the pathogenesis of several cognitive disorders, including epilepsy,^53^ autism^54^ and schizophrenia.^7, 55^ Also, many studies show that disrupted GABAergic interneuron signaling and reduced inhibitory activity are linked to these pathological conditions.^9, 56, 57^ Increasing evidence shows that abnormalities in brain extracellular matrix components, including proteoglycans, likely contribute to some aspects of pathophysiologies involving synaptic dysfunction and altered GABAergic, glutamatergic and dopaminergic transmission.^58, 59^ Interestingly, previous studies show that *Nrg1* and *Erbb4* mutant mice have schizophrenic-like deficits such as hyperactivity, impaired prepulse inhibition and latent inhibition, reduced working memory, cognitive deficits and social withdrawal.^60, 61, 62, 63, 64, 65, 66, 67, 68, 69, 70, 71^ Similarly, neuropsin-KO mice exhibit relevant behavioral deficits.^15, 72^ Although the expression levels of the *NRG1* transcript and NRG1 protein in patients with schizophrenia remain controversial, serum levels of soluble NRG1 are reportedly low in these patients,^73^ suggesting impaired release of NRG1. Altogether, these results suggest that mutations in the genes encoding neuropsin or NRG1, which inhibit the production of the ligand moiety or generate a functionally impaired ligand, will disrupt NRG1–ErbB4 signaling and alter the E/I balance, possibly leading to the cognitive dysfunctions observed in bipolar and schizophrenic syndromes.
