# Supplementary figures and images for: Ablation of neuropsin–neuregulin 1 signaling imbalances ErbB4 inhibitory networks and disrupts hippocampal gamma oscillation
Source: Transl Psychiatry. 2017 Mar 7;7(3):e1052–. doi: 10.1038/tp.2017.20 (PMC5416666; doi:10.1038/tp.2017.20)

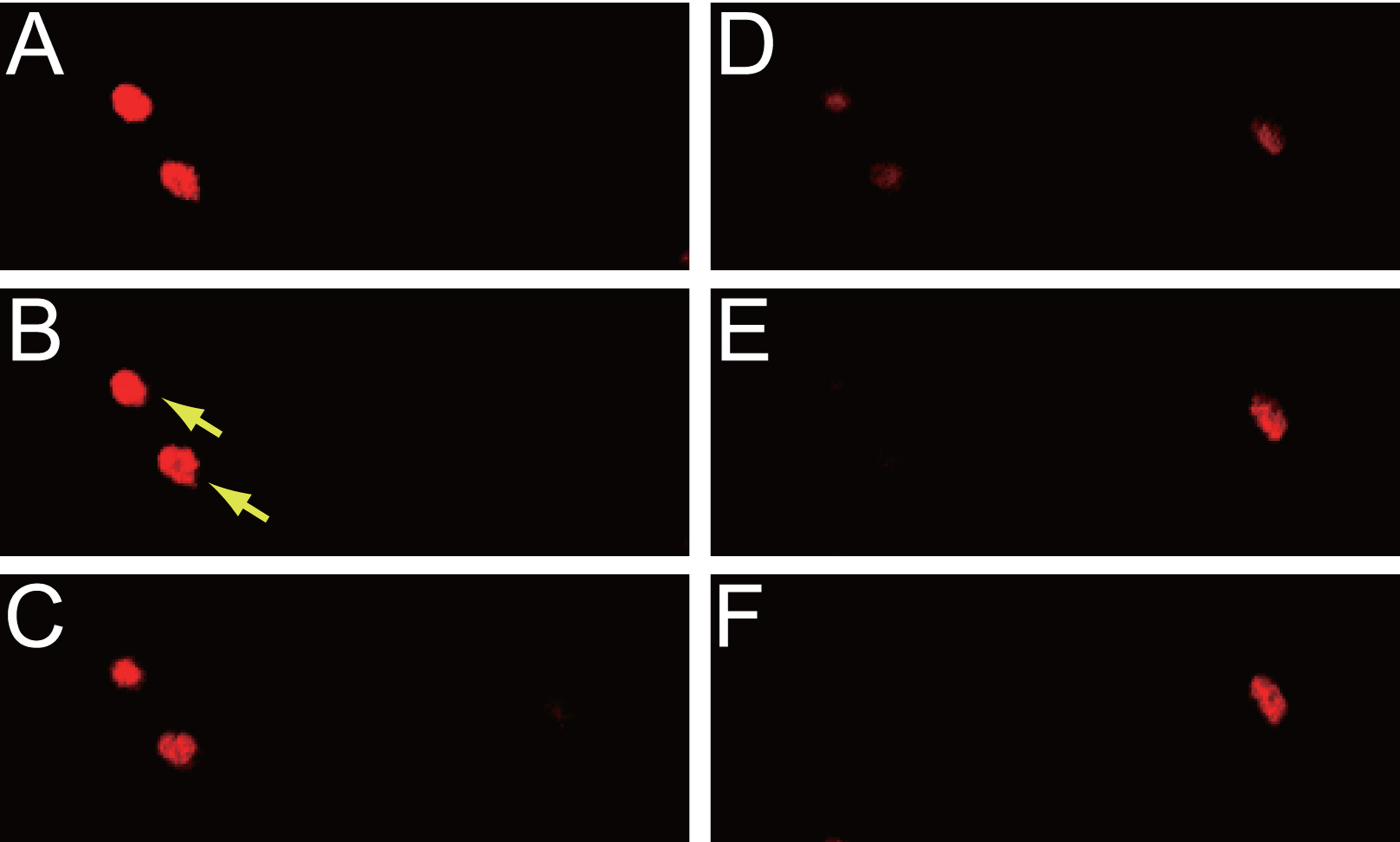

Supplement: Supplementary Figure 1 [file tp201720x1.tif]

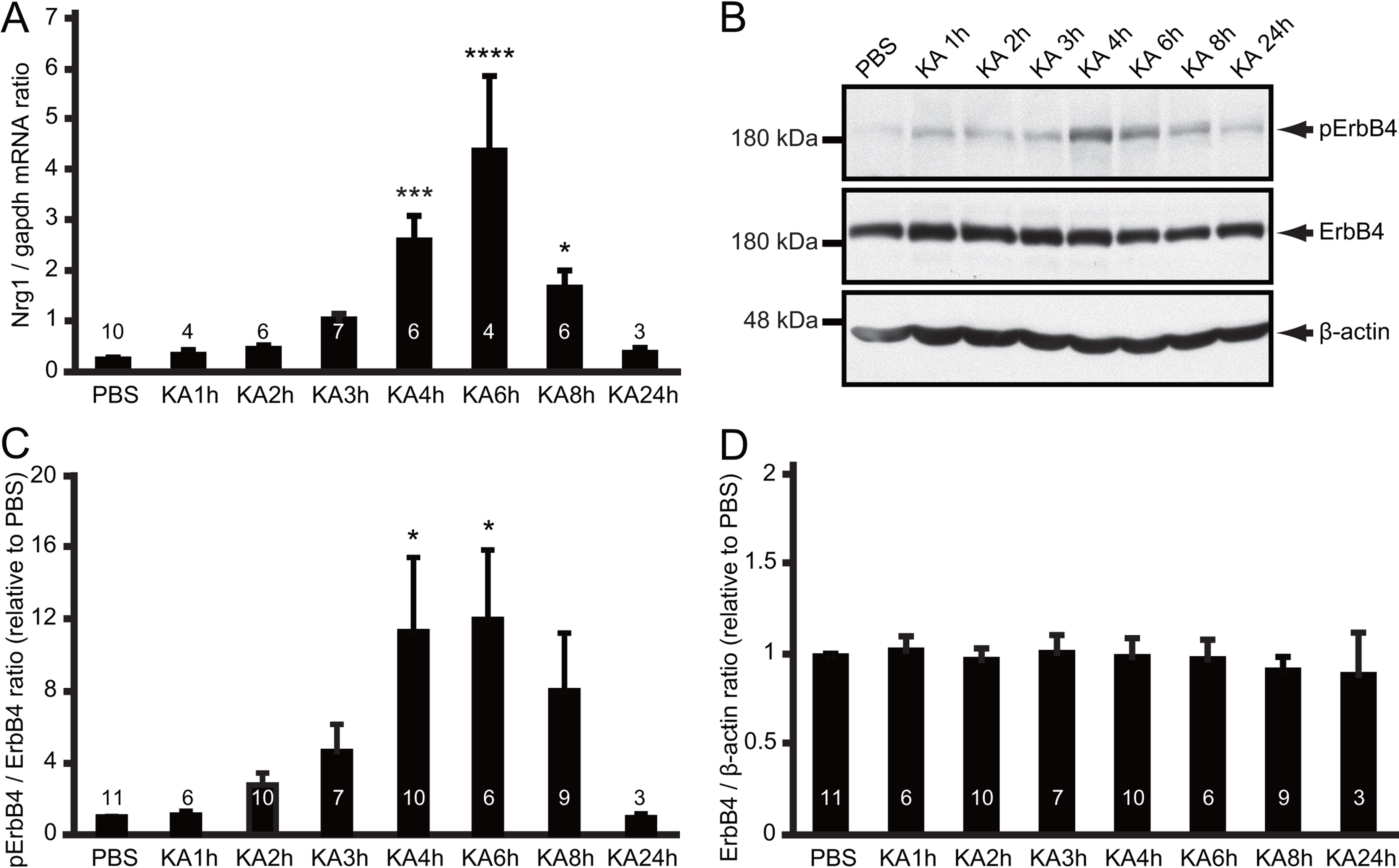

Supplement: Supplementary Figure 2 [file tp201720x2.tif]

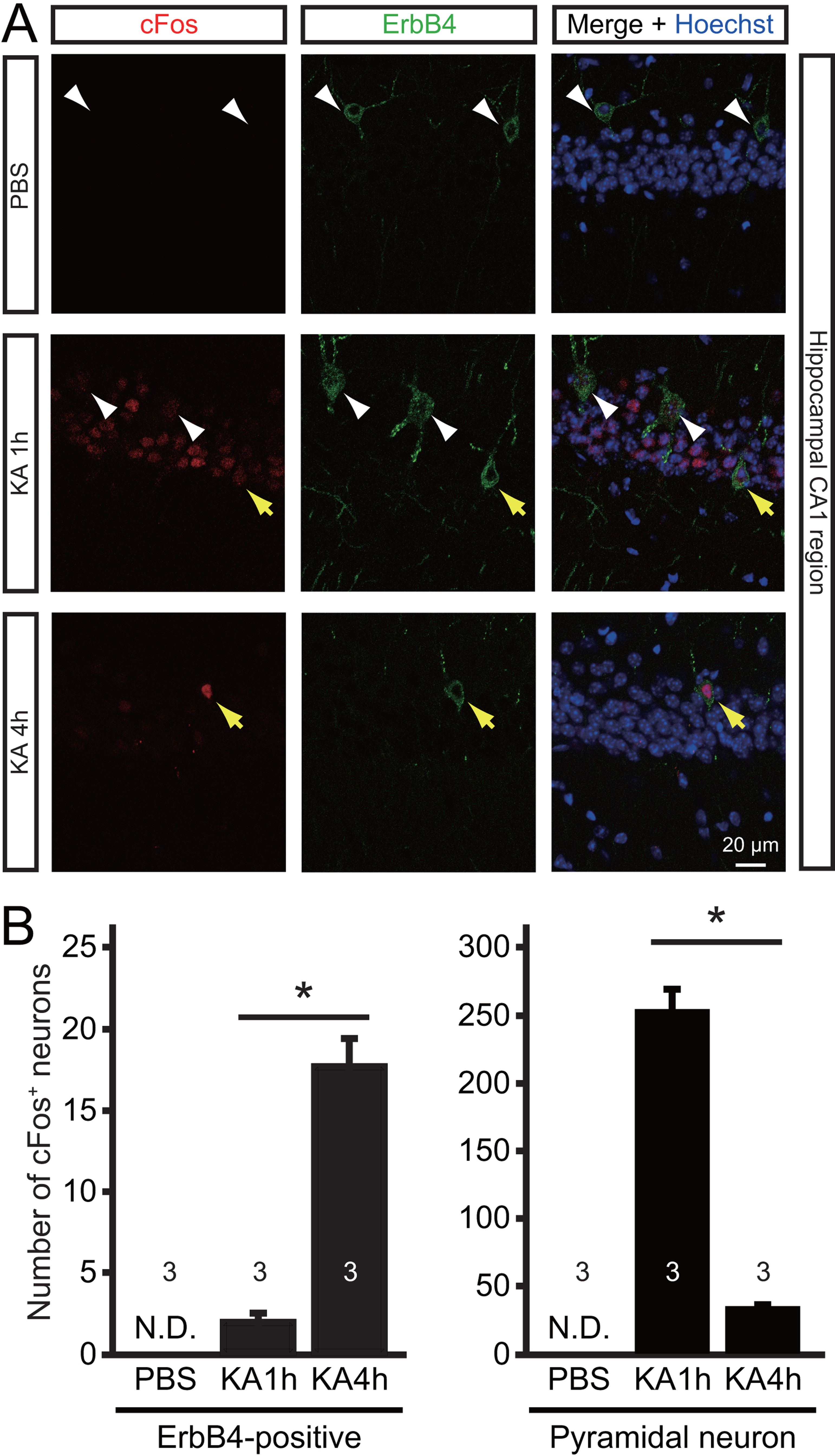

Supplement: Supplementary Figure 3 [file tp201720x3.tif]

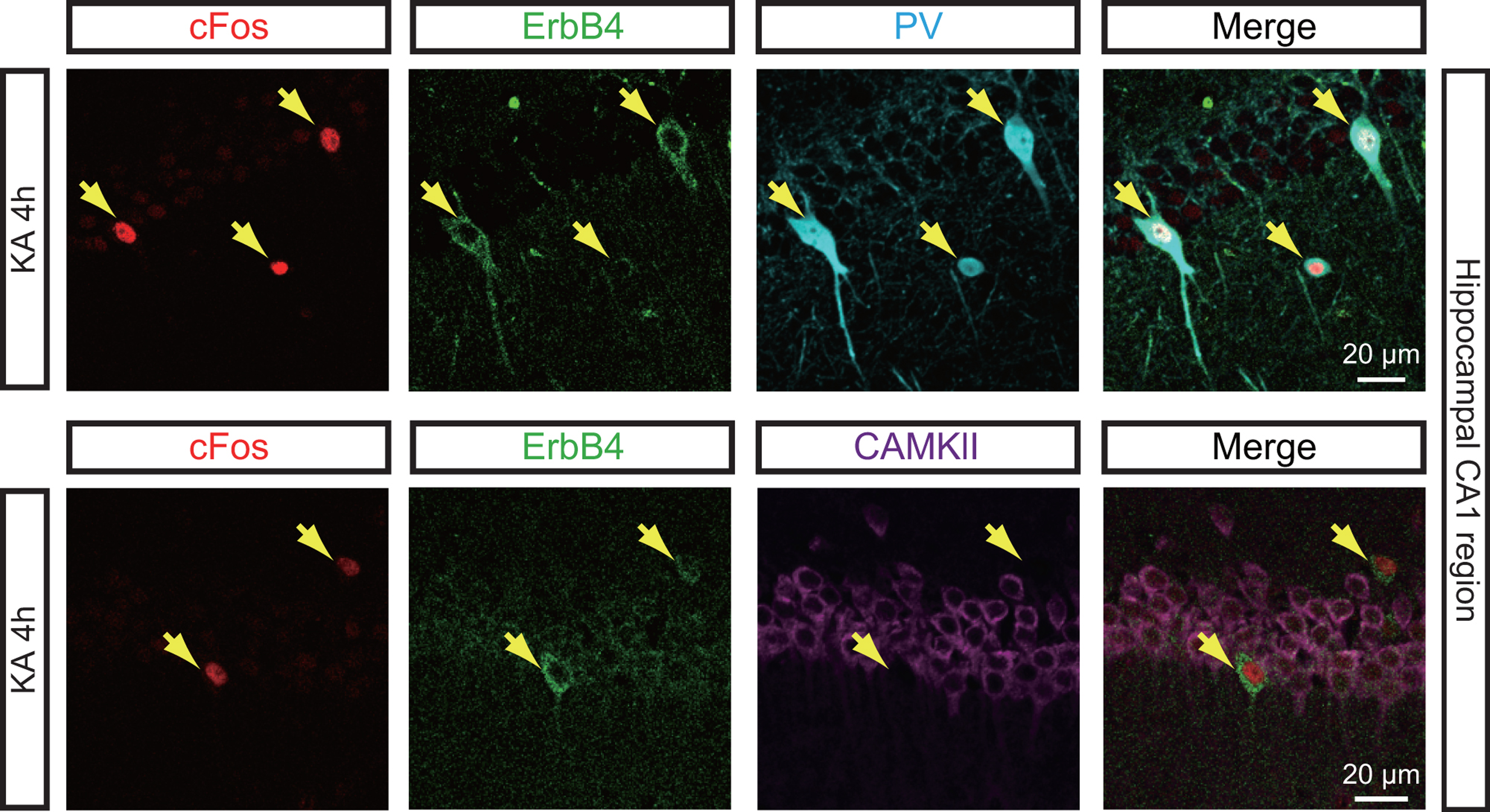

Supplement: Supplementary Figure 4 [file tp201720x4.tif]

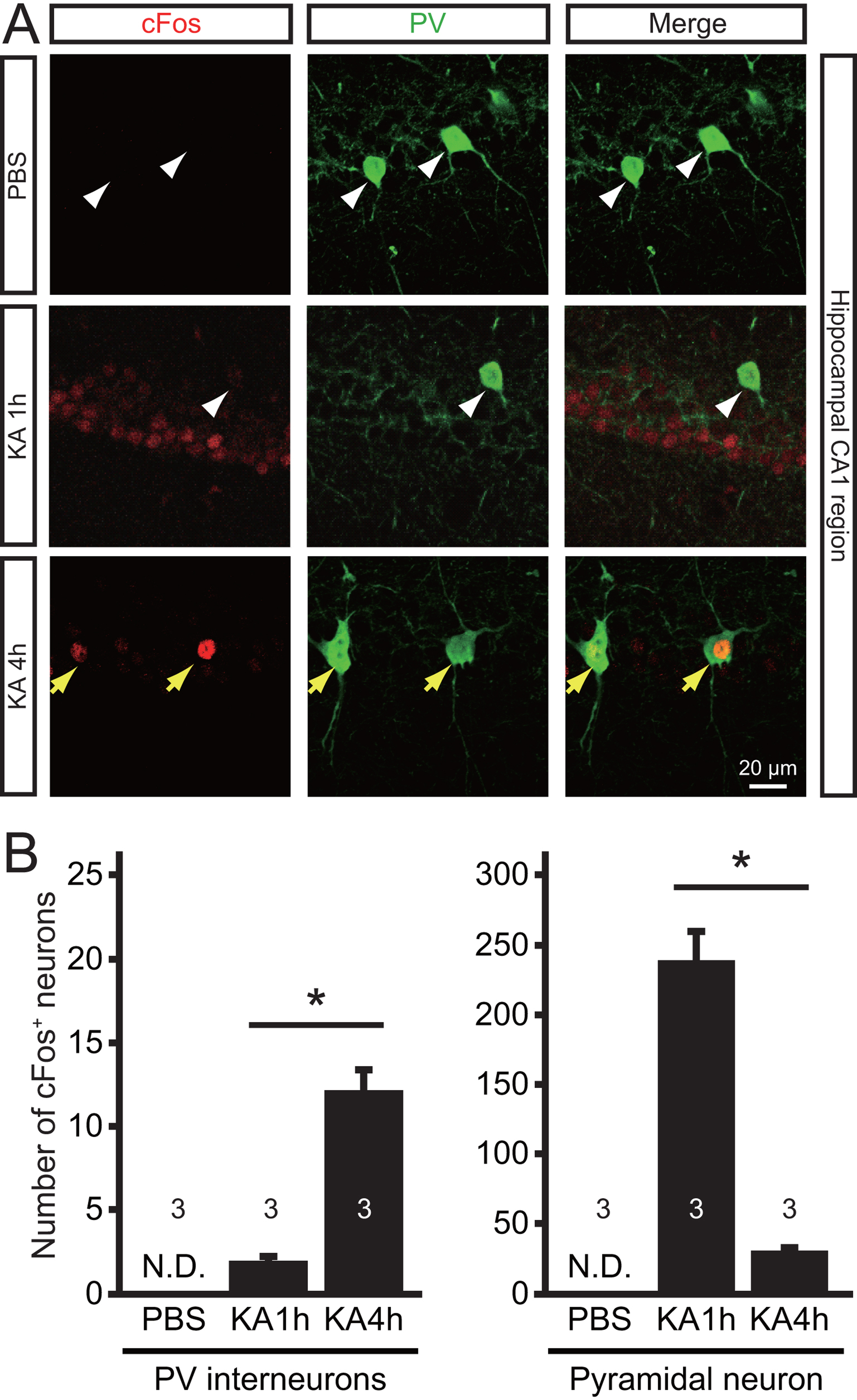

Supplement: Supplementary Figure 5 [file tp201720x5.tif]

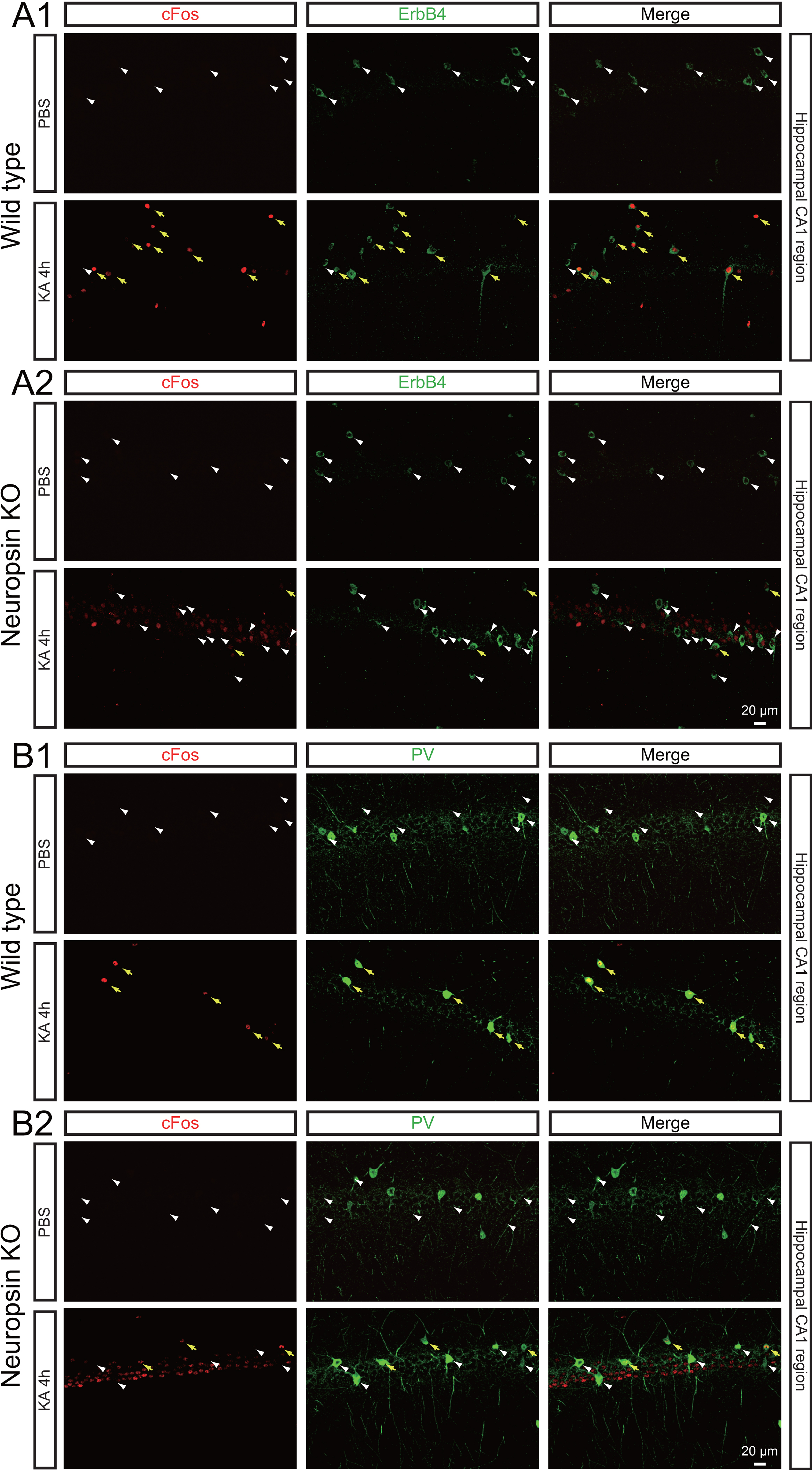

Supplement: Supplementary Figure 6 [file tp201720x6.tif]

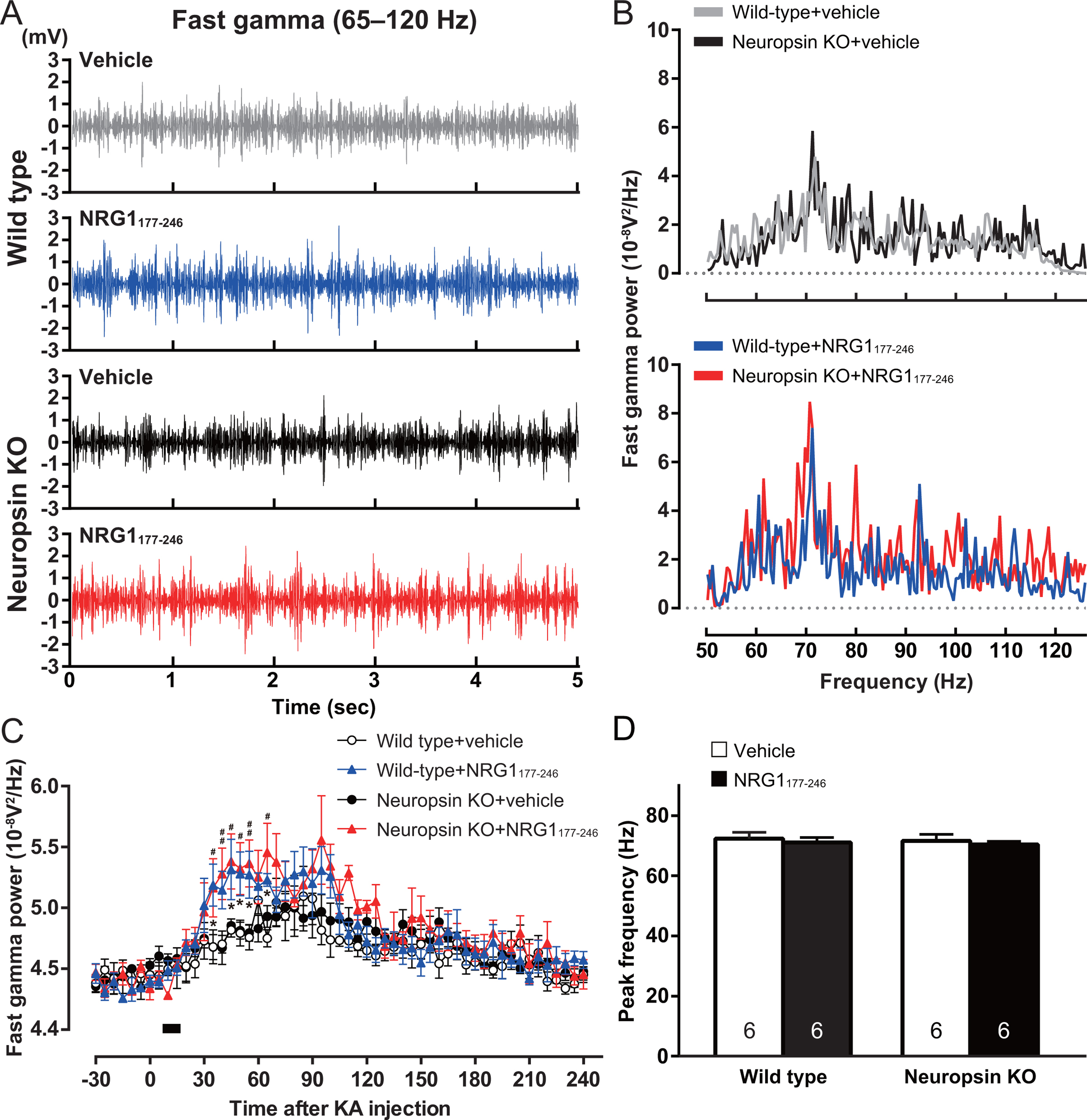

Supplement: Supplementary Figure 7 [file tp201720x7.tif]
