# Supplementary material for: Ablation of neuropsin–neuregulin 1 signaling imbalances ErbB4 inhibitory networks and disrupts hippocampal gamma oscillation
Source: Transl Psychiatry. 2017 Mar 7;7(3):e1052–. doi: 10.1038/tp.2017.20 (PMC5416666; doi:10.1038/tp.2017.20)
Supplement: Supplementary Table 1 [file tp201720x8.docx]

**Supplementary Table 1. Comparison of KA dosage and seizure onset time between wild-type and neuropsin KO mice.**

|  | **KA dosage Seizure onset time**  ***n*  (mg/kg) (min)** |
| --- | --- |
| **Wild type**  **Neuropsin KO**  ***P* value** | 11 33.18 ± 2.26 80.27 ± 10.7  11 32.27 ± 1.95 77.91 ± 8.39  0.7641 0.8634 |

The total dosage of KA used to obtain a continuous motor seizure (stage 4 or 5) and the seizure onset time until tonic-clonic movements (stage 4) were observed are calculated. Values are means ± SEM.
