# Supplementary material for: Ablation of neuropsin–neuregulin 1 signaling imbalances ErbB4 inhibitory networks and disrupts hippocampal gamma oscillation
Source: Transl Psychiatry. 2017 Mar 7;7(3):e1052–. doi: 10.1038/tp.2017.20 (PMC5416666; doi:10.1038/tp.2017.20)
Supplement: Supplementary Table 2 [file tp201720x9.docx]

**Supplementary Table 2. Overlap of markers in cFos-positive neurons in the hippocampal CA1 region 4 h after KA administration.**

|  | ***n* cFos/Marker Marker/cFos** |
| --- | --- |
| **ErbB4**  **PV**  **ErbB4+PV** | 5 68.21 ± 4.78 78.53 ± 6.70  5 75.21 ± 5.02 37.98 ± 4.36  5 81.78 ± 3.98 35.21 ± 6.10 |

Quantification analysis of cFos-positive neurons 4 h after KA administration in the hippocampal CA1 region of C57BL/6J mice that express ErbB4 or parvalbumin (PV). Percent cells expressing marker or cFos per 500,000 μm^3^ express as means ± SEM. *n* indicates the number of animals analyzed.
