# Supplementary material for: Ablation of neuropsin–neuregulin 1 signaling imbalances ErbB4 inhibitory networks and disrupts hippocampal gamma oscillation
Source: Transl Psychiatry. 2017 Mar 7;7(3):e1052–. doi: 10.1038/tp.2017.20 (PMC5416666; doi:10.1038/tp.2017.20)
Supplement: Supplementary Information [file tp201720x10.docx]

**Supplementary Figure legends**

**Figure S1. Disector-based cell counting*.***

An image gallery was created from six subsequent histological sections (2 μm apart) immunostained with anti-cFos antibody (red). The first section is shown in (A), the second one in (B), the third one in (C), the forth one in (D), the fifth one in (E), and the last one in (F). Only cells (yellow arrows) are counted if they appeared in one (reference) section (panel B in this example) but not the other (look-up) section (panel E in this example).

**Figure S2. Seizure activity induces expression of *Nrg1 type I* mRNA and activation of ErbB4 in the hippocampus *in vivo.***

(A) Changes in the relative expression of hippocampal *Nrg1 type I* mRNA at various times after KA administration measured with real-time RT-PCR. *Gadph* mRNA is used as an internal control. Hippocampal *Nrg1 type I* mRNA is increased in mice 4, 6, and 8 h after KA injection compared with that in PBS-injected control mice (one-way ANOVA, *F*_(7,38)_ = 10.15; KA 4 h, ^***^*P* = 0.0004; KA 6 h, ^****^*P* < 0.0001; KA 8 h, ^*^*P* = 0.0475 *vs.* PBS). (B) Time course of change in phosphorylated ErbB4 (pErbB4), total ErbB4, and β-actin protein levels after KA administration in mouse hippocampus. (C and D) Quantitative analysis of pErbB4 and ErbB4 using western blotting. Ratios for pErbB4/ErbB4 (C) and ErbB4/β-actin (D) are shown. The fold change was normalized to PBS in wild-type mice. pErbB4 protein, but not ErbB4 protein, levels are upregulated 4 and 6 h after KA administration (one-way ANOVA, *F*_(7,54)_ = 3.057; KA 4 h, ^*^*P* = 0.0118; KA 6 h, ^*^*P* = 0.0244 *vs*. PBS). Error bars indicate the SEM. ^*^*P* < 0.05, ^***^*P* < 0.001, and ^****^*P* < 0.0001; one-way ANOVA with Dunnett’s post-hoc test. Numbers inside columns indicate *n*.

**Figure S3. cFos activation is delayed in ErbB4-positive neurons after KA administration.**

(A) Changes in cFos (red) labeling of ErbB4-expressing neurons (green) in the hippocampal CA1 region, 1 h (middle panels) and 4 h (lower panels) after KA administration. Merged images are counterstained with Hoechst 33342 dye to visualize nuclei (blue). Yellow arrows indicate ErbB4-positive neurons expressing cFos. White arrowheads indicate ErbB4-positive neurons that do not express cFos. (B) Quantitative analysis of cFos immunofluorescent labeling in ErbB4-positive neurons (left) and pyramidal neurons (right) at 1 h and 4 h post-KA administration to the C57BL/6J mice shown in (A). ErbB4-labeled neurons show strong cFos labeling at 4 h. Mice injected with PBS (upper panel) were used as a control. N.D. indicates not detected. Error bars indicate the SEM. ^*^*P* < 0.05; Mann-Whitney *U* test. Numbers inside columns indicate *n*.

**Figure S4. ErbB4-positive neurons exhibiting delayed seizure-related activation are not excitatory neurons but are parvalbumin-expressing interneurons.**

Triple immunofluorescence images of cFos (red), ErbB4 (green) and parvalbumin (PV; cyan; upper panel) or calcium/calmodulin-dependent protein kinase (CAMKII; magenta; lower panel) in the hippocampal CA1 region 4 h after KA administration. Yellow arrows indicate that cFos/ErbB4 double labeling is detected in cells expressing parvalbumin, but not in cells expressing CAMKII.

**Figure S5. cFos activation is delayed in parvalbumin-positive interneurons after KA administration.**

(A) Changes in cFos (red) labeling of parvalbumin (PV)-positive interneurons (green) in the hippocampal CA1 region, 1 h (middle panels) and 4 h (lower panels) after KA administration. Yellow arrows indicate parvalbumin-positive interneurons expressing cFos. White arrowheads indicate parvalbumin-positive interneurons that do not express cFos. (B) Quantitative analysis of cFos immunofluorescent labeling in parvalbumin (PV)-positive interneurons (left) and pyramidal neurons (right) at 1 h and 4 h post-KA administration to the C57BL/6J mice shown in (A). Parvalbumin-positive interneurons show strong cFos labeling at 4 h. Mice injected with PBS (upper panels) were used as a control. N.D. indicates not detected. Error bars indicate SEM. ^*^*P* < 0.05; Mann-Whitney *U* test. Numbers inside columns indicate *n*.

**Figure S6. Neuropsin-KO mice show low cFos expression in ErbB4-positive neurons and parvalbumin-positive interneurons after seizure.**

Representative low-magnification images showing cFos expression (red) in ErbB4-positive neurons (green; A1 and A2), parvalbumin-positive interneurons (green; B1 and B2), and pyramidal neurons in wild-type (A1 and B1) or neuropsin-KO (A2 and B2) mice at 4 h after administration of PBS (upper panel) or KA (lower panel). Yellow arrows indicate ErbB4-positive neurons (A1 and A2) or parvalbumin-positive interneurons (B1 and B2) expressing cFos. White arrowheads indicate ErbB4-positive neurons (A1 and A2) or parvalbumin-positive interneurons (B1 and B2) that do not express cFos. Fluorescent labeling of the hippocampal CA1 region revealed that ErbB4-positive neurons and parvalbumin-positive interneurons in wild-type mice show more number of neurons stained for cFos than those in neuropsin-KO mice.

**Figure S7. Normal fast gamma oscillations in neuropsin-KO mice.**

(A and B) Representative local field potential traces (65–120 Hz) (A) and power spectra (B) of KA-induced fast gamma oscillations in wild-type mice after injection of vehicle (grey) or NRG1_177-246_ (blue) and in neuropsin-KO mice after injection of vehicle (black) or NRG1_177-246_ (red). (C) Time course for the mean (and SEM) power (65–120 Hz) of KA-induced fast gamma oscillations in wild-type mice after injection of vehicle (white circles; *n* = 6 mice) or NRG1_177-246_ (blue triangles; *n* = 6 mice) and in neuropsin-KO mice after injection of vehicle (black circles; *n* = 6 mice) or NRG1_177-246_ (red triangles; *n* = 6 mice). Differences in the power of KA-induced fast gamma oscillations were observed in the CA1 region after injection of vehicle or NRG1_177-246_ (*F*_(3,20)_ = 0.6128, *P* = 0.6146; two-way ANOVA). Similar effects were observed after KA administration to both wild-type and neuropsin-KO mice injected with NRG1_177-246_. (^*^*P* < 0.05 *vs*. vehicle-injected wild-type mice, and ^#^*P* < 0.05 and ^##^*P* < 0.01 *vs*. vehicle-injected neuropsin-KO mice; two-way ANOVA with the Sidak post-hoc test). NRG1_177-246_ or vehicle was injected during the time period indicated by the bar. (D) Bar graph showing the mean (and SEM) peak frequency of KA-induced fast gamma oscillations in wild-type mice and neuropsin-KO mice after injection of vehicle (white bar) or NRG1_177-246_ (black bar). There was no difference in the peak frequency of slow gamma oscillations between the groups (*F*_(3,20)_ = 0.2076, *P* = 0.8899; one-way ANOVA with Tukey’s post-hoc test). Numbers inside columns indicate *n*.
